# Global analysis of double-strand break processing reveals *in vivo* properties of the helicase-nuclease complex AddAB

**DOI:** 10.1371/journal.pgen.1006783

**Published:** 2017-05-10

**Authors:** Anjana Badrinarayanan, Tung B. K. Le, Jan-Hendrik Spille, Ibrahim I. Cisse, Michael T. Laub

**Affiliations:** 1 Department of Biology, Massachusetts Institute of Technology, Cambridge, MA, United States of America; 2 National Centre for Biological Sciences (NCBS), Tata Institute of Fundamental Research, Bangalore, India; 3 Department of Molecular Microbiology, John Innes Centre, Norwich, United Kingdom; 4 Department of Physics, Massachusetts Institute of Technology, Cambridge, MA, United States of America; 5 Howard Hughes Medical Institute, Massachusetts Institute of Technology, Cambridge, MA, United States of America; National Cancer Institute, UNITED STATES

## Abstract

In bacteria, double-strand break (DSB) repair via homologous recombination is thought to be initiated through the bi-directional degradation and resection of DNA ends by a helicase-nuclease complex such as AddAB. The activity of AddAB has been well-studied *in vitro*, with translocation speeds between 400–2000 bp/s on linear DNA suggesting that a large section of DNA around a break site is processed for repair. However, the translocation rate and activity of AddAB *in vivo* is not known, and how AddAB is regulated to prevent excessive DNA degradation around a break site is unclear. To examine the functions and mechanistic regulation of AddAB inside bacterial cells, we developed a next-generation sequencing-based approach to assay DNA processing after a site-specific DSB was introduced on the chromosome of *Caulobacter crescentus*. Using this assay we determined the *in vivo* rates of DSB processing by AddAB and found that putative *chi* sites attenuate processing in a RecA-dependent manner. This RecA-mediated regulation of AddAB prevents the excessive loss of DNA around a break site, limiting the effects of DSB processing on transcription. In sum, our results, taken together with prior studies, support a mechanism for regulating AddAB that couples two key events of DSB repair–the attenuation of DNA-end processing and the initiation of homology search by RecA–thereby helping to ensure that genomic integrity is maintained during DSB repair.

## Author summary

Double-strand breaks (DSBs) are a threat to genome integrity and are faithfully repaired via homologous recombination. The initial processing of DSB ends that prepares them for recombination has been well-studied *in vitro*, but is less well characterized *in vivo*. We describe a deep sequencing-based assay for assessing the early steps of DSB processing in bacterial cells by the helicase-nuclease complex AddAB. We find that a combination of *chi* site recognition and RecA loading is required to attenuate AddAB activity. In the absence of RecA, the chromosome is excessively degraded with a concomitant loss in transcription. Our results, along with prior studies, support a model for how *chi* recognition and RecA together regulate AddAB to maintain genome integrity and facilitate recombination.

## Introduction

Double-strand breaks (DSBs) are a potentially lethal form of DNA damage as incorrectly repaired or unrepaired breaks can lead to the loss of genetic information, chromosomal rearrangements, mutations, or cell death. Cells have evolved the ability to faithfully repair DSBs via homologous recombination using a sister chromatid or sister chromosome as a template [1–3]. In all domains of life, homologous recombination requires the processing of DSB ends to produce single-stranded DNA overhangs [3–5]. In bacteria this processing is carried out by a helicase-nuclease complex, such as AddAB or RecBCD, whereas eukaryotes use multiple complexes including the Rad50/Mre11 complex [3,4]. The single-stranded DNA overhangs produced by the helicase-nuclease complex become bound by a single-stranded DNA-binding recombinase, usually RecA in bacteria, or the homologous Rad51 in eukaryotes. The RecA/Rad51 filaments that form on ssDNA overhangs can initiate homology search and strand invasion to drive recombination and subsequent repair of the damaged chromosome [1–3].

Precisely how DSB end processing occurs and how it is regulated to ensure the generation of single-stranded DNA required for recombination without an excessive loss of genomic information is not fully understood. Biochemical studies have led to two general models. In one model, the nuclease-helicase complex initially degrades both strands of DNA until it encounters a specific DNA element called *chi* (*c*rossover *h*otspot *i*nstigator) that triggers a switch to a state that drives resection of only one strand, thereby producing the necessary ssDNA overhang needed to initiate homologous recombination [6–13]. This model has emerged from biochemical studies on *E*. *coli* RecBCD, which has two independent helicase domains and a nuclease domain, and *B*. *subtilis* AddAB, which contains a helicase and a nuclease domain in AddA along with a nuclease domain in AddB (which also carries an inactive helicase domain) [4,5,14]. Biochemical studies of AddAB from *B*. *subtilis* show that, like RecBCD, recognition of *chi* sequences on the 3’-terminated strand during degradation converts AddAB from a double-stranded nuclease to a single-stranded DNA nuclease and slows its effective rate of translocation [15–18]. Structural studies have further suggested that this could be due to a conformational change in the complex following *chi* recognition by AddB [14,19]. The single-stranded DNA generated by either RecBCD or AddAB is thought to be bound by the RecA filament. In the case of RecBCD, the loading mechanism for RecA has been well-characterized, showing that RecA binding to single-stranded DNA is facilitated by RecBCD in a *chi-*dependent manner, via a direct interaction with RecB [8,20,21]. How RecA loading occurs in the context of AddAB remains to be determined. The alternative model for production of single-stranded overhangs posits, at least in *E*. *coli*, that RecBCD initially unwinds a DSB end but without any degradation [6]. Upon activation at a *chi* site RecBCD then nicks one strand with subsequent helicase activity separating the two strands to create a single-stranded overhang that can load RecA and initiate homologous recombination.

Which of these two models applies *in vivo* to AddAB is not fully resolved. As noted, the molecular events underlying the initial processing of DSBs have been extensively studied *in vitro*, both in bulk and in single-molecule experiments [14–17,19,22–24], but assessing these events and measuring the rates of AddAB or RecBCD-dependent processing of a DSB on the chromosome in living cells remains a major challenge. Genetic assays used to assess RecBCD [25,26], and to some extent AddAB [27–30] activity, *in vivo* have provided important insights, but direct measurements of DNA processing by these helicase-nuclease complexes has been limited. The assays used often involve measuring the retention of radioactively labeled nucleotides in chromosomes subjected to UV damage [31], which produces a large number of lesions and kills most cells, complicating the estimation of degradation rates by a helicase-nuclease complex acting on a single chromosomal DSB. Techniques such as Southern blotting [32,33] have also been used to probe helicase-nuclease activity *in vivo*, but cannot easily be used to examine DNA processing on a global level. Advances in whole genome DNA sequencing in combination with the development of systems for the controlled introduction of single DSBs [32,34–36] in bacterial chromosomes now offer the ability to probe the *in vivo* activity of helicase-nuclease complexes like AddAB with higher resolution and more precision.

*Caulobacter crescentus* is a useful organism for probing the *in vivo* dynamics and mechanisms underlying DSB repair. Unlike many bacteria, *Caulobacter* exhibits once-and-only-once replication of its chromosome under all growth conditions, and large populations of cells are easily synchronized, enabling the isolation and study of cells with a single chromosome. DSB repair has been previously visualized at the single-cell level in *Caulobacter* [37]. Here, we examine the *in vivo* processing of site-specific DSBs introduced in the *Caulobacter* chromosome, which is thought to require AddAB [37,38], using the endonuclease I-SceI [34,37]. Using a deep sequencing-based assay we measure the extent of DNA processing by AddAB around a break site and provide evidence that AddAB initially degrades both strands, but is then triggered, by putative *chi* sites, to resecting a single strand. We show that putative *chi* sites in *Caulobacter* attenuate the rate of AddAB-mediated DNA processing *in vivo*, but only with ~20% efficiency, similar to *in vitro* estimates for *B*. *subtilis* AddAB [16]. We find that, in the absence of RecA, AddAB translocation rates inferred *in vivo* are comparable to the previously measured *in vitro* rate of ~400 bp/s for *B*. *subtilis* AddAB [16,22,23,39]. Further, our results suggest that, in the presence of RecA, AddAB translocation after *chi* recognition is reduced ~4-fold. Successful attenuation of degradation requires the formation of a RecA filament, but not the SOS response or recombination. Collectively, our results indicate that RecA likely downregulates the translocation rate of AddAB after *chi*, possibly through a direct protein-protein interaction. This regulation of AddAB by RecA helps to limit DNA degradation around a break site, thus constraining the impact on transcription to a more limited region of the genome.

## Results

### A deep sequencing-based assay for measuring DNA resection after a double-strand break

**Fig 1:**
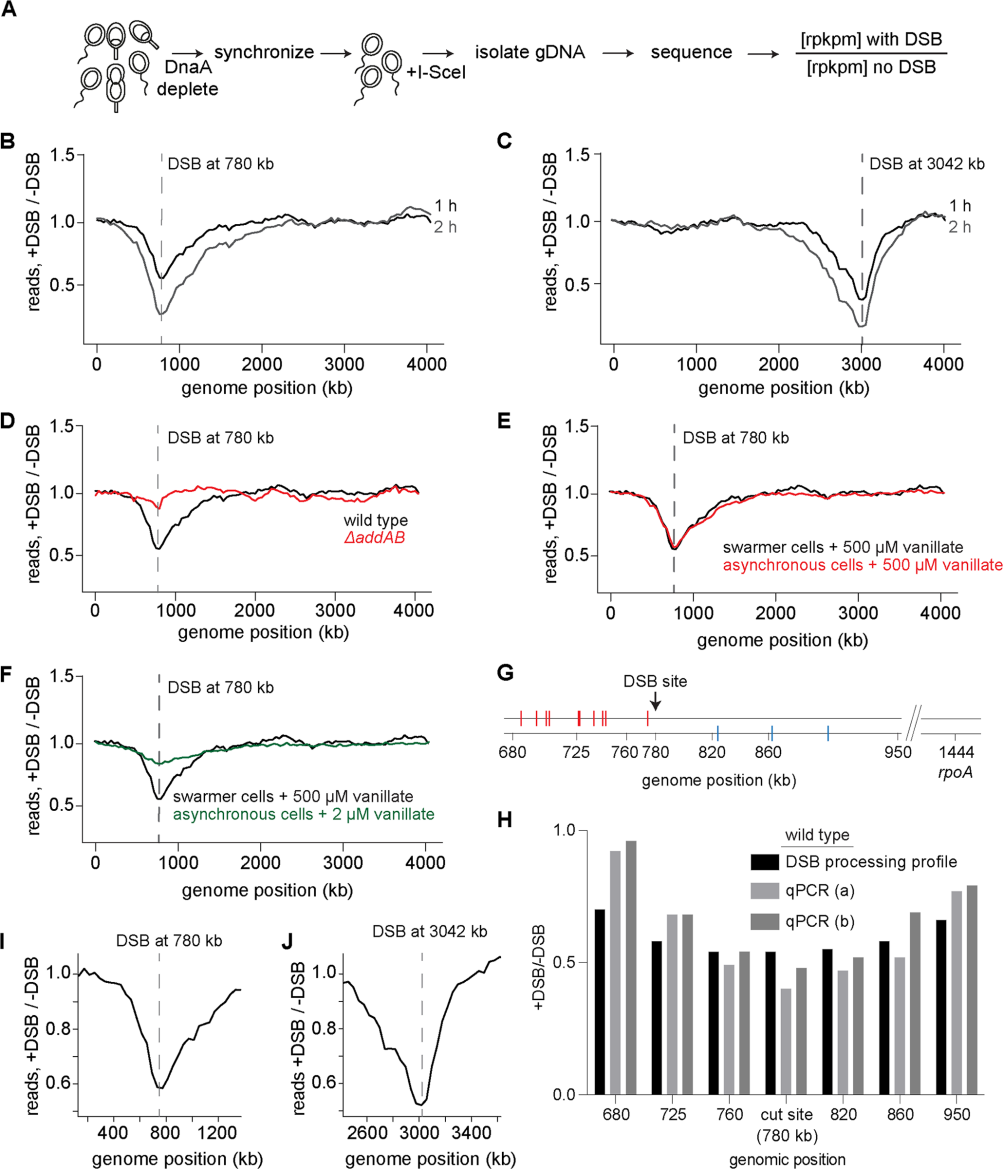
A deep sequencing-based assay for measuring DNA processing after a double-strand break. (A) Schematic of the assay used to measure DNA processing around a DSB in *Caulobacter* swarmer cells. DSBs were induced by addition of 500 μM vanillate. (B-C) Representative DSB processing profiles for DSBs induced at +780 (B) or +3042 (C) kb for 1 h (black) or 2 h (grey). Location of the DSB site is indicated with a dashed line. (D) Representative profile showing that, in the absence of AddAB (red trace), DNA processing and degradation around a DSB site is not observed. (E) Profiles for DSB at +780 kb in an asynchronously growing population of *Caulobacter* induced with 500 μM (red) vanillate for 1 h. Profile for DSB induction in swarmer cells with 500 μM vanillate from Fig 1B is also shown in black. (F) Profiles for DSB at +780 kb in an asynchronously growing population of *Caulobacter* induced with 2 μM (green) vanillate for 1 h. Profile for DSB induction in swarmer cells with 500 μM vanillate from Fig 1B is also shown in black. (G-H) qPCR was performed using the primer pairs at the genomic positions indicated in wild-type cells. The location of putative *chi* sites on the top and bottom strands are indicated with red and blue, respectively, tick marks (also see Fig 2A). Samples were collected before (0 min) and 60 min after DSB induction. qPCR at a distal, unprocessed control site (*rpoA*: +1,444 kb) was also performed. qPCR values at each locus normalized to *rpoA* are plotted for two independent repeats (a and b). DSB processing profile values for the same loci are also shown. (I-J) Zoomed in regions of the 1 h profiles for a DSB induced at +780 (I) or +3042 kb (J).

To assess DNA processing around a DSB induced on the chromosome, we used the I-SceI system previously developed in *Caulobacter* [37] (Fig 1A). Briefly, a single I-SceI site was introduced +780 or +3042 kb from the origin of replication and the I-SceI enzyme, which recognizes and cleaves the I-SceI site to generate a DSB, was placed under a vanillate-regulated promoter on the chromosome [37,40]. This promoter is repressed by the protein VanR; addition of vanillate releases VanR from the P_*van*_ promoter and induces gene expression [40]. DnaA, the replication initiator, was expressed from an IPTG inducible promoter to control the replication state of cells. To isolate the initial step of DSB processing from later events of homologous recombination, we conducted our experiments primarily in cells with a single chromosome, the swarmer cells of a *C*. *crescentus* population. To isolate these swarmer cells and to prevent subsequent rounds of replication, cells were grown without IPTG for 1.5 h to deplete DnaA and arrest cells in a G1 state. These G1-arrested cells were then isolated using Percoll density gradient centrifugation [41]; flow cytometry analysis verified that this procedure produced a population of G1-phased swarmer cells (S1A Fig). I-SceI was then induced by adding vanillate for 0.5, 1, 2, or 4 h and genomic DNA isolated and sequenced. As a control, genomic DNA from swarmer cells to which vanillate was not added was also isolated and sequenced. The fold difference in reads per kilobase per million (rpkpm) in the DSB-induced sample relative to the control was plotted as a function of genomic position, hereafter referred to as a DSB processing profile.

In cells with a single chromosome, induction of a DSB with 500 μM vanillate at +780 kb from the origin resulted in bidirectional loss of DNA around the break site, with an ~30% and ~40% drop in reads at the DSB site after 0.5 h and 1 h respectively (Fig 1B, S1B and S1C Fig). After 2 h, there was an ~60% drop in reads at the location of the DSB site. Similar decreases in reads were also observed upon induction of a DSB +3042 kb from the origin (Fig 1C, S1D Fig). In this case, 1 h of vanillate induction resulted in an ~60% drop in reads at the site of the DSB with 2 h of induction resulting in an ~80% drop in reads. The DSB processing profiles were highly reproducible, with independent repeats yielding r values of 0.94 (S1E Fig).

To confirm that the troughs observed in the profiles (Fig 1B and 1C) result from DSB processing by AddAB, we repeated our assay in *ΔaddAB* cells. These cells did not show any significant drop in reads near the DSB site, or elsewhere in the genome (Fig 1D), indicating that AddAB is, in the growth conditions tested here, the only helicase-nuclease complex that processes a DSB.

We also generated a DSB processing profile for an unsynchronized, actively replicating population of cells. We first treated an asynchronous population of cells with 500 μM vanillate (as with the synchronized G1/swarmer cells in Fig 1B) to drive maximal induction of I-SceI, which is sufficient to drive cleavage of both chromosomes in nearly all cells with two chromosomes [37], thereby preventing homologous recombination-based repair. However, even with maximal induction of I-SceI, a very small percentage of cells could experience only a single DSB that can be repaired. Treatment with 500 μM vanillate produced a profile almost identical to that observed with swarmer cells alone (Fig 1E). This effect was not because cells entered a G1 arrest (S1G Fig), indicating that AddAB activity is not significantly influenced by the replication status of the cells. We also measured the DSB processing profile for cells in which I-SceI was induced with 2 μM vanillate (Fig 1F). These cells likely experience only a single DSB and thus can repair the damaged chromosome through homologous recombination-based repair, as judged by the fact that cells treated with 2 μM vanillate showed no major change in their flow cytometry profile (S1G Fig), did not lose viability, and were previously shown to engage in homology-based repair [37]. The profile for these cells was similar in shape to that of synchronized swarmer cells or asynchronous cells treated with 500 μM vanillate, but the magnitude of differences was substantially reduced (Fig 1F), likely because some fewer cells experience DSBs and because cells can repair a single cut chromosome.

The library preparation procedure used should, in principle, only result in the sequencing of double-stranded DNA. However, DSB resection could, in principle, result in the production of some single-stranded DNA. To ensure that we were not sequencing single-stranded DNA, we treated genomic DNA extracted from a DSB-induced sample with Mung bean nuclease, which degrades single-stranded DNA. The resulting degradation profile was indistinguishable from that of a sample not treated with the nuclease (S1F Fig), indicating that our method only produces reads for double-stranded DNA. Thus, our profiles likely represent total DNA loss due to the degradation of one or both strands from the DSB. This interpretation would also fit with prior biochemical studies indicating that AddAB can degrade double-stranded DNA and resect a single strand. However, as noted before, an alternative model for *E*. *coli* RecBCD posits that the helicase-nuclease complex initially unwinds the two strands and then, at *chi* sites will nick and again unwind but not degrade the DNA. If the initially unwound DNA reannealed, it would form double-stranded DNA that would be sequenced. But if that were occurring, we would not have seen any loss of reads near the DSB site. Alternatively, the initially unwound DNA may not anneal, remaining single-stranded, which is not captured in our sequencing. Hence, to distinguish between these possibilities, we performed quantitative PCR (qPCR), which can report on total DNA, including any single-stranded DNA that may be missed in our sequencing assay. If AddAB were only unwinding the DNA flanking a DSB, then qPCR using primer pairs at loci adjacent to a DSB should yield significantly more product than a primer pair that spans the DSB site itself. However, the qPCR values for loci immediately adjacent to a DSB site at +780 kb were comparable to the value for the DSB site itself, and to the values measured by our sequencing-based profiling, supporting the notion that AddAB initially degrades both strands of DNA (Fig 1G and 1H). The qPCR values increased at sites further from the DSB site, relative to the qPCR value at the DSB and relative to the values measured by our sequencing approach, likely reflecting the presence of some single-stranded DNA not detected in our sequencing assay. Thus, taking together prior biochemical studies and our own sequencing and qPCR data, we favor a model in which AddAB initially degrades both strands and then, in response to *chi* sites (see below), switches to resecting a single strand. There is also a formal possibility that AddAB does initially just unwind the DSB end and that other nucleases in the cell degrade each strand, but such a model is less parsimonious as it invokes additional components that are not necessary *in vitro*.

### DNA degradation around a double-strand break is asymmetric due to putative *chi* sites

In the 1 h DSB processing profile for swarmer cells with a DSB induced at +780 kb (Fig 1B) the read counts were lowest at the site of cleavage and then increased progressively in both directions until they matched the read counts of the control profile. These profiles can, therefore, be used to estimate an upper bound on the speed of AddAB-dependent processing. After 1 h, the first point of separation between the DSB-induced and control profiles was ~450 kb to the left of the DSB and ~1300 kb to the right. Thus, the rate of processing (degradation and resection) *in vivo* can be, at most, ~100 bp/s to the left and ~200 bp/s to the right. However, because the profiles are not step functions and instead feature a gradual decrease from +450 and +1300 kb toward the DSB site, it implies that most cells degrade DNA more slowly than 100–200 bp/s or initiate DSB processing at different times. To test this latter possibility, we measured cell viability as a function of time after adding vanillate to induce I-SceI and a DSB. Because we induce DSBs in swarmer cells containing a single chromosome, recombination-based repair cannot occur and a DSB is lethal. Thus, if all cells experienced a DSB immediately after the addition of vanillate, we would expect a precipitous drop in viability post-induction. Instead, we found a gradual decrease in viability suggesting that cells likely experience a DSB at variable times (S1H Fig). Heterogeneity in a population of cells may also arise if DNA degradation proceeds at different rates in individual cells or if DSB processing is slowed or stopped at different frequencies, possibilities explored further below.

**Fig 2:**
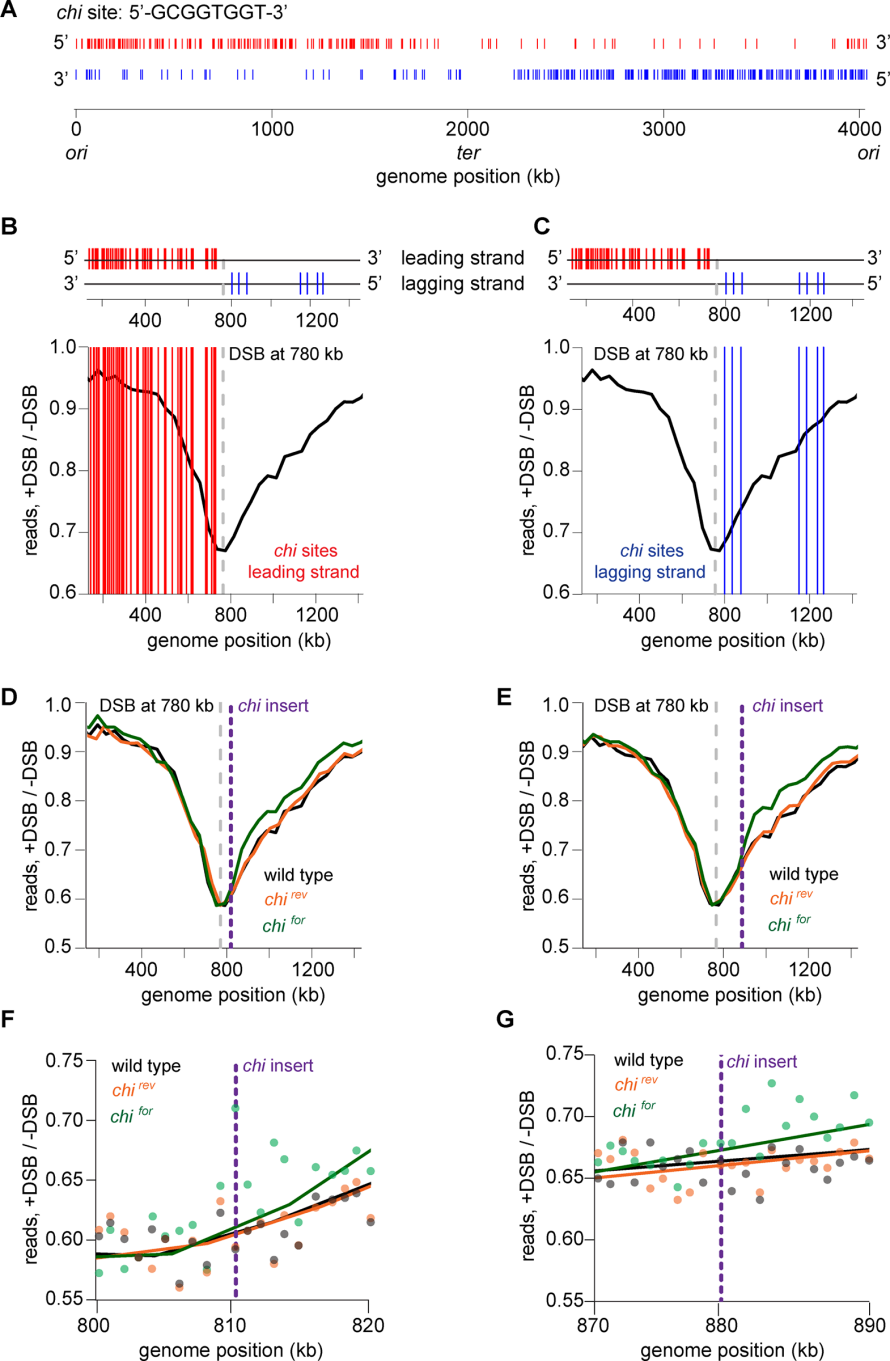
DNA degradation around a double-strand break is asymmetric due to chi sites. (A) Plot showing distribution of the putative *chi* sequence (5’-GCGGTGGT-3’) in *Caulobacter*. *chi* sites that are predicted to have an effect on AddAB translocation on the leading strand are shown in red and on the lagging strand are shown in blue. Each tick indicates the position of an individual *chi* sequence. (B-C) Positions of *chi* sequences on the leading strand (B) or lagging strand (C) with respect to a DSB site at +780 kb (dashed line) are overlaid on the DSB processing profile from Fig 1E. (D) A repeat of 15 *chi* sequences (*chi*^*for*^) was inserted +30 kb from the DSB site at +780 kb and the profile is shown in green. As a control, the *chi* orientation was flipped at the same location (*chi*^*rev*^) and the profile is shown in orange. Profile of a DSB induced at +780 kb (without insertion of *chi*) is also shown from Fig 1B (black). (E) Same as panel D, with *chi* sequences inserted +100 kb from the DSB site. (F-G) Zoom in views of genomic regions where *chi* sequences were inserted, as in panels D-E.

For DSBs at either +780 or +3042 kb, the global processing profile was clearly asymmetric around the break site (Fig 1I and 1J). In each case, read loss extended further toward the terminus than the origin. Prior studies have shown that DNA degradation by helicase-nuclease complexes such as AddAB or RecBCD is negatively regulated by *chi* sequences that are highly abundant in bacterial genomes, and often with a much higher frequency on the lagging-strand template, with respect to DNA replication [2,42]. Because *B*. *subtilis* AddAB directionally recognizes *chi* sites [4], the distribution of these sequences may underlie the asymmetry seen in our degradation profiles. The putative *chi* sequence in *Caulobacter* was previously predicted computationally to be 5’-GCGGTGGT-3’ [43] (Fig 2A). To test whether this sequence element is responsible for degradation asymmetry, we first overlaid on the DSB processing profile of cells with a DSB induced at +780 kb the putative *chi* sequences on the leading (Figs 2B and S2A) and lagging strands (Figs 2C and S2B) that may affect AddAB translocation and degradation in the 3’->5' direction. Leading and lagging strands are defined with respect to DNA replication, which is presumed to proceed bidirectionally from the origin (0 kb) to the terminus (~2000 kb); for the sake of simplicity, we use 'leading' and 'lagging' strands to refer to the lagging- and leading-strand templates, respectively (Fig 2A). Additionally, we note that the *chi* sequences shown in Fig 2 and throughout our study are those that match the computationally predicted *chi* sequence and are in an orientation that would, in principle, allow them to affect AddAB. The presence of putative *chi* sequences was inversely correlated with the extent of degradation, with more degradation occurring on the side of the DSB where the density of putative *chi* sequences was substantially less. This pattern was also observed when a DSB was induced +3042 kb from the origin (S2C and S2D Fig) suggesting that the asymmetry observed in the DSB processing profiles results from an effect of *chi* site frequency on AddAB activity.

To more directly determine whether 5’-GCGGTGGT-3’ is the *Caulobacter chi* sequence and whether the presence of this sequence explains the asymmetry of our profiles, we inserted an array of 15 such sequences on the chromosome either +30 kb or +100 kb from the DSB site at +780 kb. This *chi* array was inserted either in the correct orientation for recognition by AddAB (*chi*^*for*^) or in the opposite orientation as a control (*chi*^*rev*^). After inducing a DSB, the *chi*^*rev*^ construct had no effect on degradation (Fig 2D and 2E, S2E and S2F Fig). In contrast, the *chi*^*for*^ construct significantly reduced degradation beyond the location it was inserted when compared to the control or wild-type profiles (Figs 2D, 2E, S2E and S2F). Although degradation beyond the inserted array of *chi* sites was reduced, it was not completely eliminated. Given prior studies suggesting that *chi* sites switch AddAB to a mode in which it degrades only one strand to produce resected DNA [4,17], we infer that ssDNA degradation likely occurs more slowly than the initial double stranded degradation. Alternatively, it is possible that the *chi* recognition frequency is low. These data also support the conclusion that 5'-GCGGTGGT-3' is likely the *chi* sequence in *Caulobacter*, though we have not, of course, shown whether they are sites where homologous recombination preferentially occurs. Importantly for our purposes though, these sites clearly affect DSB processing and likely explain the asymmetric degradation by AddAB from a DSB site.

Notably, the *chi*^*for*^ construct produced a clear difference in the profile, relative to the wild type and cells harboring the *chi*^*rev*^ construct. This difference was evident within ~2–5 kb of the site of insertion (Fig 2F and 2G), demonstrating that our assay has a resolution of at least 5 kb and likely better.

### Effect of *chi* sites on AddAB is dependent on RecA

**Fig 3:**
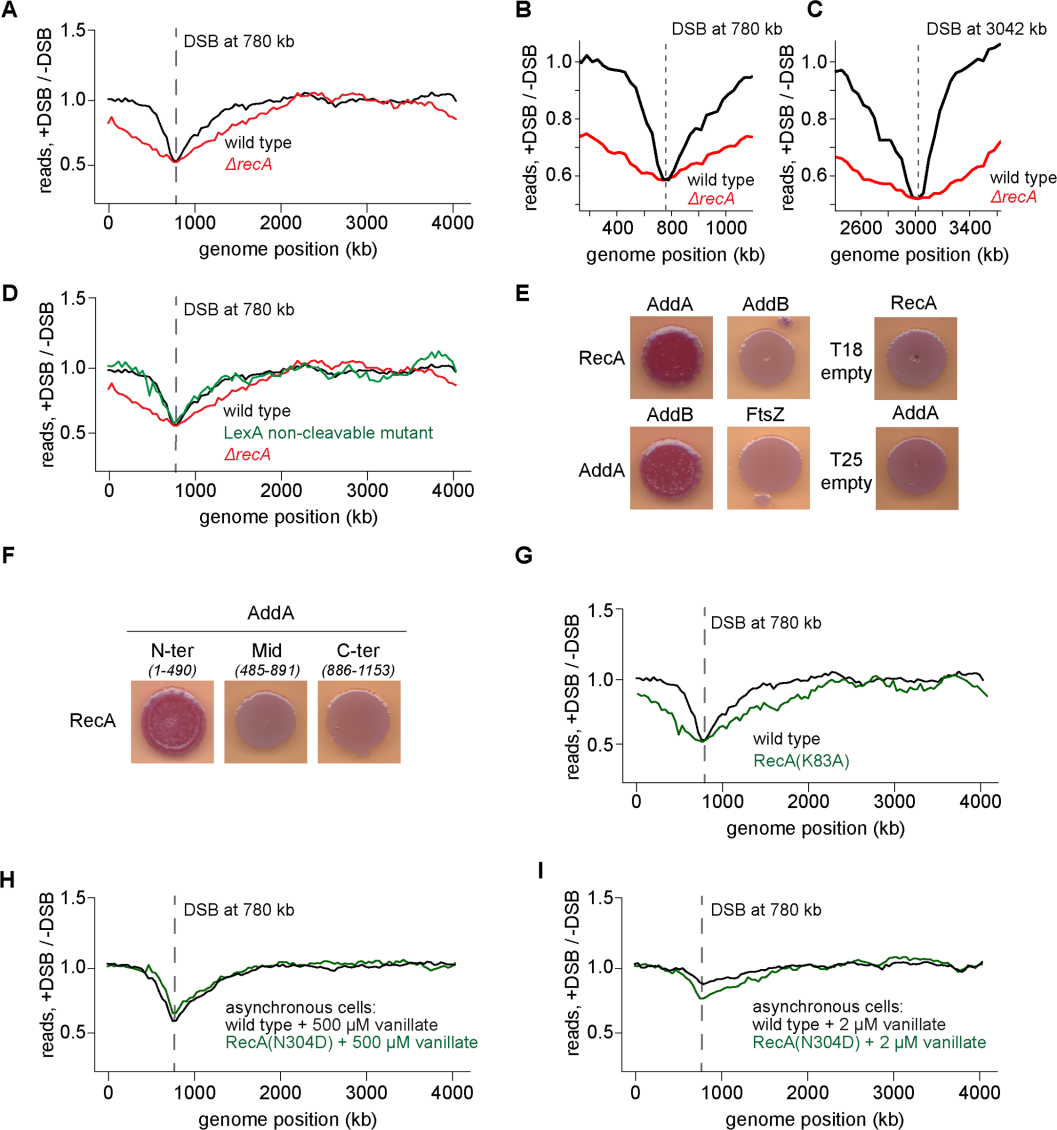
Effect of chi sites on AddAB is dependent on RecA. (A) Representative DSB processing profile for a DSB induced at +780 kb from the origin 1 h after I-SceI induction with (black) or without (red) RecA. Location of the DSB site is indicated with a dashed line. The wild-type profile is from Fig 1B. (B) Zoomed in profile for a DSB induced at +780 kb 1h after I-SceI induction with (black) or without (red) RecA. (C) As (B) for a DSB induced at +3042 kb. (D) Profile of a non-cleavable mutant of LexA (green) is shown. Profiles of wild type (black) and *ΔrecA* (red) are from Fig 3A. (E) Bacterial-two-hybrid assay showing RecA interaction with AddA, but not AddB. Empty vector controls are also shown. (F) Bacterial-two-hybrid assay with fragments of AddA. Amino acid positions for each fragment are indicated in parentheses. (G) DSB processing profile of a predicted RecA ATPase mutant, RecA(K83A) (green). The wild type profile (black) is from Fig 1B. (H) Profile of a predicted RecA recombination deficient mutant, RecA(N304D) (green). The wild-type profile (black) is from Fig 1E. In both cases, asynchronously growing, replicating cells were induced with 500 μM vanillate for 1h. (I) Same as panel H but for asynchronously growing, replicating cells induced with 2 μM vanillate for 1 h.

Collectively, the results presented thus far suggest that the recognition of *chi* sequences by AddAB results in an attenuation of AddAB-mediated processing of DNA around a break site. Next, we wondered whether the attenuation of DSB end processing after *chi* recognition may result, in part, from the recruitment of RecA to the ssDNA formed after AddAB encounters a *chi* site. To examine the effect of RecA on DSB processing, we measured the DSB processing profile of cells lacking RecA (Fig 3A, S3C and S3D Fig). In sharp contrast to the wild type, the profile for Δ*recA* cells exhibited significantly more extensive degradation and/or processing in both directions after a DSB (Fig 3A). Additionally, the asymmetry of the degradation profile was no longer apparent in cells lacking RecA (Fig 3B and 3C). After 1 h, the first point of separation between the DSB-induced and control profiles was approximately the same on both sides of the DSB, yielding an upper bound of ~400 bp/s for the rate of DNA processing in both directions in *ΔrecA* cells. Note that we also detected more total DNA via qPCR than in our DSB processing profiles of *ΔrecA* cells, again with a progressively increasing ratio of qPCR to degradation profile values away from the DSB site (S3A and S3B Fig). These results indicate that RecA is important in limiting the extent of DSB end processing.

The effect of RecA on DSB resection could be indirect. RecA, when bound to single-stranded DNA, can induce auto-cleavage of the transcriptional repressor LexA, resulting in the expression of genes in the SOS regulon, many of which participate in DNA damage response and repair [44,45]. To test whether the effect of RecA on AddAB-dependent degradation around a DSB is indirectly mediated via the SOS response, we introduced a non-cleavable mutant of LexA [46] into our DSB system and measured the profile of cells after a DSB. The profile for this non-cleavable LexA mutant strain was nearly indistinguishable from the wild-type profile (Fig 3D). Thus, the effect of RecA on AddAB-dependent degradation is likely not mediated through its effect on the SOS regulon. Further, the dispensability of the SOS response for AddAB regulation suggests that basal levels of RecA are sufficient to prevent excessive DNA processing at a DSB [45].

To test whether RecA directly interacts with AddAB, we used a bacterial two-hybrid assay to screen for physical interactions [47]. Each protein was fused to a subunit of adenylate cyclase and then co-expressed in *E*. *coli*. Interaction between two fusion proteins will reconstitute adenylate cyclase, leading to production of cAMP and the subsequent activation of a reporter gene that turns colonies red on MacConkey agar. In this assay, we found that RecA interacted with AddA but not with AddB (Figs 3E, S3E and S3F). We also confirmed an interaction between AddA and AddB, as expected, but not between AddA and a negative control, FtsZ. RecA or AddA also did not display interaction with empty vector controls, T18 and T25 respectively. Further, our assay indicated that RecA likely interacts with the N-terminal portion of AddA (Figs 3F, S3E and S3F), where the helicase domain of AddA resides. This is in contrast to RecA’s interaction with RecB in *E*. *coli*, which occurs via the nuclease domain of RecB [20].

RecA forms a filament on single-stranded DNA that is formed by AddAB after it interacts with *chi* and begins degrading only one strand of the DNA [2,4,5]. To test whether this filament forming activity of RecA is necessary for it to interact with and regulate AddAB, we generated a mutant, RecA(K83A), predicted to abrogate filament formation based on studies of *E*. *coli* RecA [48–50]. This mutant retained an interaction with AddA in the bacterial two-hybrid system and was expressed *in vivo* at levels comparable to the wild type protein (S3G Fig). However, the K83A mutant was incapable of regulating AddAB-mediated DNA resection as the degradation profile of the mutant looked similar to that of *ΔrecA* cells (Fig 3G). We conclude that RecA likely must form a filament to properly regulate AddAB and attenuate DNA degradation and processing.

To test whether recombination is required for AddAB regulation, or if RecA filament formation is sufficient to slow down AddAB after *chi* recognition, we constructed a strain producing RecA(N304D) [51], which is predicted to be recombination deficient but still capable of binding ssDNA to form a filament. This mutant was also expressed *in vivo* (S3G Fig), but sensitive to DSBs, comparable to *ΔrecA* cells (S3H Fig). In asynchronously growing, replicating cells induced with maximal levels of I-SceI (500 μM vanillate), the profile of this mutant was comparable to wild-type cells, including the same asymmetry around the DSB site (Fig 3H). In cells treated with only 2 μM vanillate (a concentration that allows for repair via homologous recombination in wild-type cells), an asymmetric global profile was still seen in the recombination-deficient mutant (Fig 3I). These results suggest that recombination is likely not required for RecA-mediated regulation of AddAB.

### AddAB-dependent DNA resection is attenuated after *chi* recognition

**Fig 4:**
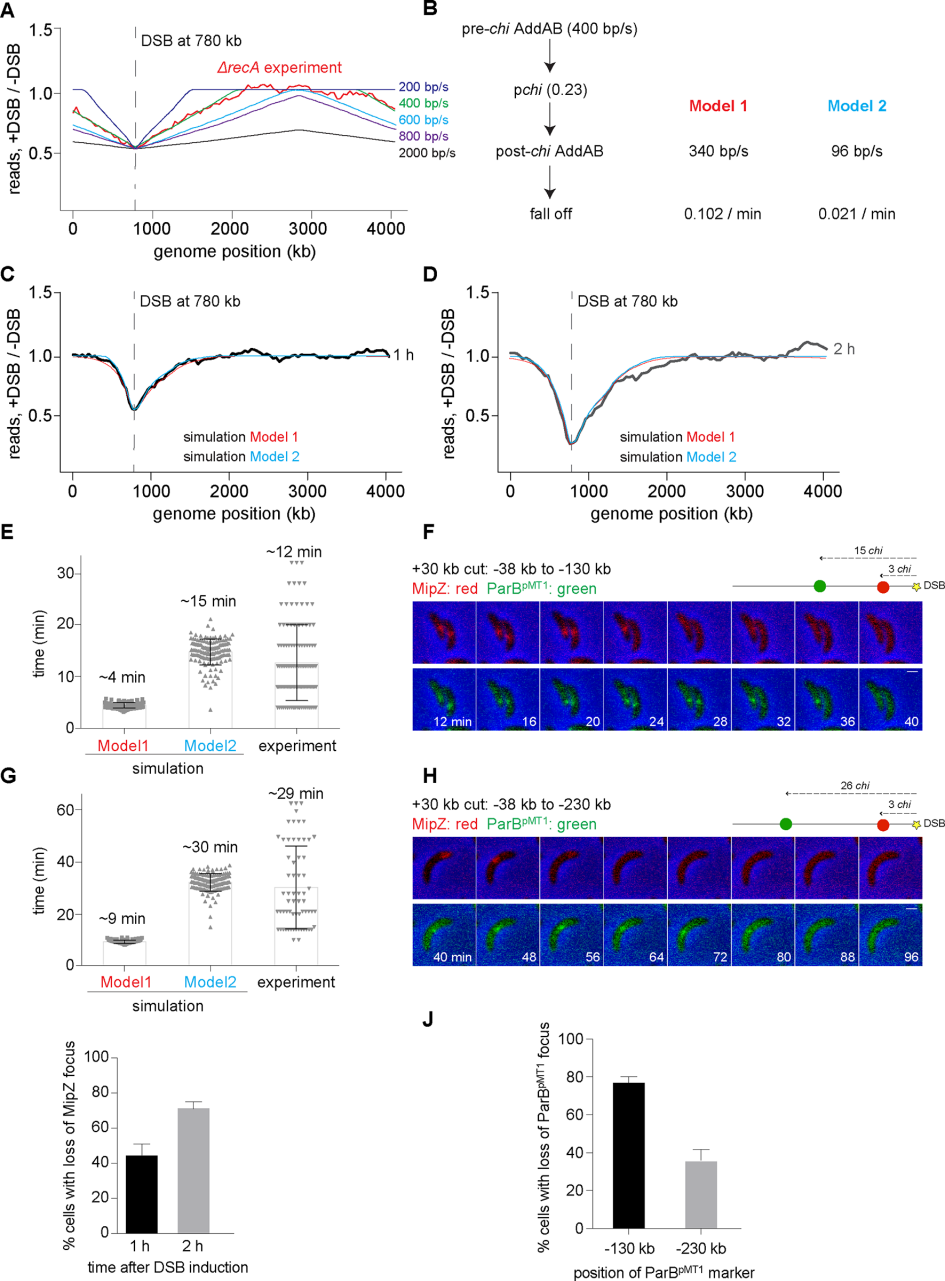
AddAB-dependent DNA processing is attenuated after chi recognition. (A) Example simulation profiles obtained by scanning for parameters that match the measured *ΔrecA* profile. The translocation rates for *B*. *subtilis* AddAB *in vitro* [16,24,39] are in green (pre-*chi* AddAB degradation rate– 400 bp/s) and black (pre-*chi* AddAB degradation rate– 2000 bp/s). In all cases, the *chi* recognition probability was ~0.23 and post-*chi* AddAB speed was reduced by 15%. The average profile from simulations of 10,000 independent cells treated for 1 h is shown. The *ΔrecA* profile (red) is from Fig 3A. (B) Schematic summarizing the possible models and parameters for describing the *in vivo* profiles of wild-type cells. (C-D) Simulation profiles obtained for Model 1 (red) and 2 (blue) that best fit the *in vivo* DNA degradation profiles for a DSB induced at +780 kb for 1 h and 2 h samples respectively. Experimental profiles from Fig 1 are overlaid in black and grey. (E) Histograms showing the predicted degradation rates from simulations of Model 1 and 2, and the experimentally obtained degradation rates between fluorescent loci at -38 kb and -130 kb for a DSB induced +30 kb from the origin. Each grey dot represents the value determined from an individual cell. The mean is reported above each bar. (F) Montage of representative cells showing the loss of fluorescent foci at -38 kb (MipZ, red) and -130 kb (ParB^pMT1^, green) after a DSB was induced +30 kb from the origin. Time is indicated in the frames. Schematic of DSB site (yellow) along with the location of the -38 kb and -130 kb markers are shown and number of *chi* sites between the DSB site and these positions is indicated. (G-H) Same as panels E-F, but for fluorescent loci at -38 kb and -230 kb for a DSB induced +30 kb from the origin. (I) Percentage of cells with the loss of a MipZ focus (-38 kb from the DSB site) 1 or 2 h after break induction. (J) Percentage of cells with loss of a ParB^pMT1^ marker -130 kb or -230 kb from the DSB site in those cells where the MipZ focus has been lost.

To further probe the effect of RecA on AddAB, we sought to assess the rates of AddAB-dependent processing *in vivo*, both before and after *chi* recognition. These rates have been measured previously *in vitro* for *B*. *subtilis* AddAB, although only in the absence of RecA [16,22,24,39]; the *B*. *subtilis* AddAB translocation rate before *chi* recognition was reported to be between 400 and 2000 bp/s, with a *chi* recognition probability of ~0.25, and a post-*chi* translocation rate only ~15% slower than the pre-*chi* rate. To estimate the *in vivo* rates and *chi* recognition probability, we ran a simulation of DNA degradation/processing with four parameters: (i) the rate of DSB formation after adding inducer, which produces a distribution of times post-induction when DSB processing begins, (ii) the degradation rate before *chi* recognition, (iii) the probability of recognizing each *chi* site, and (iv) the DNA processing rate after *chi* recognition. Rates of DSB formation were estimated via simulations (S4A and S4B Fig) and confirmed by performing qPCR on chromosomal DNA isolated from DSB-induced Δ*addAB* cells (S4C and S4D Fig). Based on previous studies [22,39], it was assumed that AddAB would recognize only one *chi* sequence and, once bound to *chi*, the complex would not be affected by further *chi* sequences encountered. With a rate of DSB formation of ~0.4 DSB / h, we first tested the parameters measured *in vitro* and found that a pre*-chi* degradation rate of 400 bp/s, a *chi* recognition probability of ~0.23, and a post-*chi* degradation rate of 340 bp/s, produced a close fit to the *in vivo* symmetric DSB processing profile of cells lacking *recA* (Fig 4A).

We next considered the case of wild-type cells in which RecA attenuates AddAB-dependent degradation and combines with *chi* to produce an asymmetric degradation profile. A reasonable fit to the wild-type profile at 1 and 2 h was produced by simulations with a pre-*chi* degradation rate of 400 bp/s, a *chi* recognition probability of ~0.22, and a post-*chi* processing rate of 51 bp/s. However, these parameters did not fit the measured profile at 4 h (S5A Fig). At this later time point, the model predicted more extensive degradation than was observed. In fact, the profile at 4 h was not substantially different than that observed at 2 h. This could indicate that cells are dead after 4 hours, although DNA degradation can likely still occur even when cells are no longer viable as judged by plating assays. Thus, the similarity between the 2 and 4 h profiles could suggest that AddAB dissociates from the DNA at long time points. We therefore added a fifth parameter to the model, the rate of AddAB dissociation and considered two possible models: Model 1 where we (i) fixed the post-*chi* processing rate to be 15% less than the pre-*chi* rate, as measured *in vitro* in the absence of RecA [16,24], and (ii) varied the rate of AddAB dissociation after *chi* recognition; Model 2 where we varied both the post-*chi* processing rate and the dissociation rate for AddAB (Fig 4B). For each model we identified parameters that produced good fits to the degradation profiles at each time point measured (Fig 4C and 4D, S5B and S5D Fig). In each case the rate of degradation pre-*chi* recognition was 400 bp/s and the probability of *chi* recognition was ~0.23. In Model 1 where the post-*chi* processing rate was 340 bp/s, the dissociation rate for AddAB was 0.102 / min. In Model 2, the post-*chi* processing rate was 96 bp/s with a dissociation rate for AddAB of 0.021 / min.

To distinguish between these models, we sought to directly measure the post-*chi* processing rate, which differs more than 3-fold between the two models, by examining the time it takes AddAB to degrade two loci positioned at specific distances from a DSB site on the arm where *chi* site density is highest. For these experiments we used a strain with a DSB site located +30 kb from the origin, which enabled us to label the endogenous *parS* locus ~38 kb from the DSB site by expressing a fusion of the protein MipZ and YFP. MipZ binds ParB, which forms a large nucleoprotein complex at *parS*; thus, MipZ-YFP forms a fluorescent focus *in vivo* that marks the cellular position of *parS* [52]. We also inserted an orthogonal *parS* site from the plasmid pMT1 either 130 or 230 kb from the break site; expressing the cognate ParB^pMT1^ fused to CFP enables the *in vivo* tracking of this locus [53]. Using time-lapse microscopy we then measured the timing of disappearance of the MipZ-YFP and ParB^pMT1^-CFP foci after inducing a DSB. We infer that the disappearance of each focus reflects the degradation of either one or both strands that correspond to a given locus, as occurs during DSB processing. Mere translocation of a protein, such as AddAB, past a locus would not lead to the permanent losses in fluorescent foci seen here; for instance, MipZ foci are well known to be maintained after the passage of the replisome [52].

The model with a post-*chi* degradation rate of 340 bp/s (Model 1 in Fig 4B) predicted an interval between loss of the two fluorescent foci of ~4 or 9 min, respectively, whereas the model with a post-*chi* degradation rate of 96 bp/s (Model 2 in Fig 4B) predicted an interval of ~15 or 30 min, respectively (Fig 4E–4J). Our measurements, using fluorescence time-lapse microscopy, revealed mean intervals of ~12 and 29 min (Fig 4E–4H), respectively, depending on whether the second locus was 130 or 230 kb from the break site. As predicted from the above results, we also found that the frequency of loss of a marker -130 kb from the break site was higher than a marker -230 kb away (Fig 4J). Thus, we favor a model in which AddAB initially drives DSB ends processing at a rate of ~400 bp/s, with an ~23% chance of recognizing each *chi* site during translocation, and that *chi* recognition in combination with RecA loading on the single-stranded DNA produced by AddAB slows subsequent processing or translocation ~4-fold, with an additional, modest rate of AddAB dissociation.

### DSB processing and degradation affects transcription of genes near a break site

**Fig 5:**
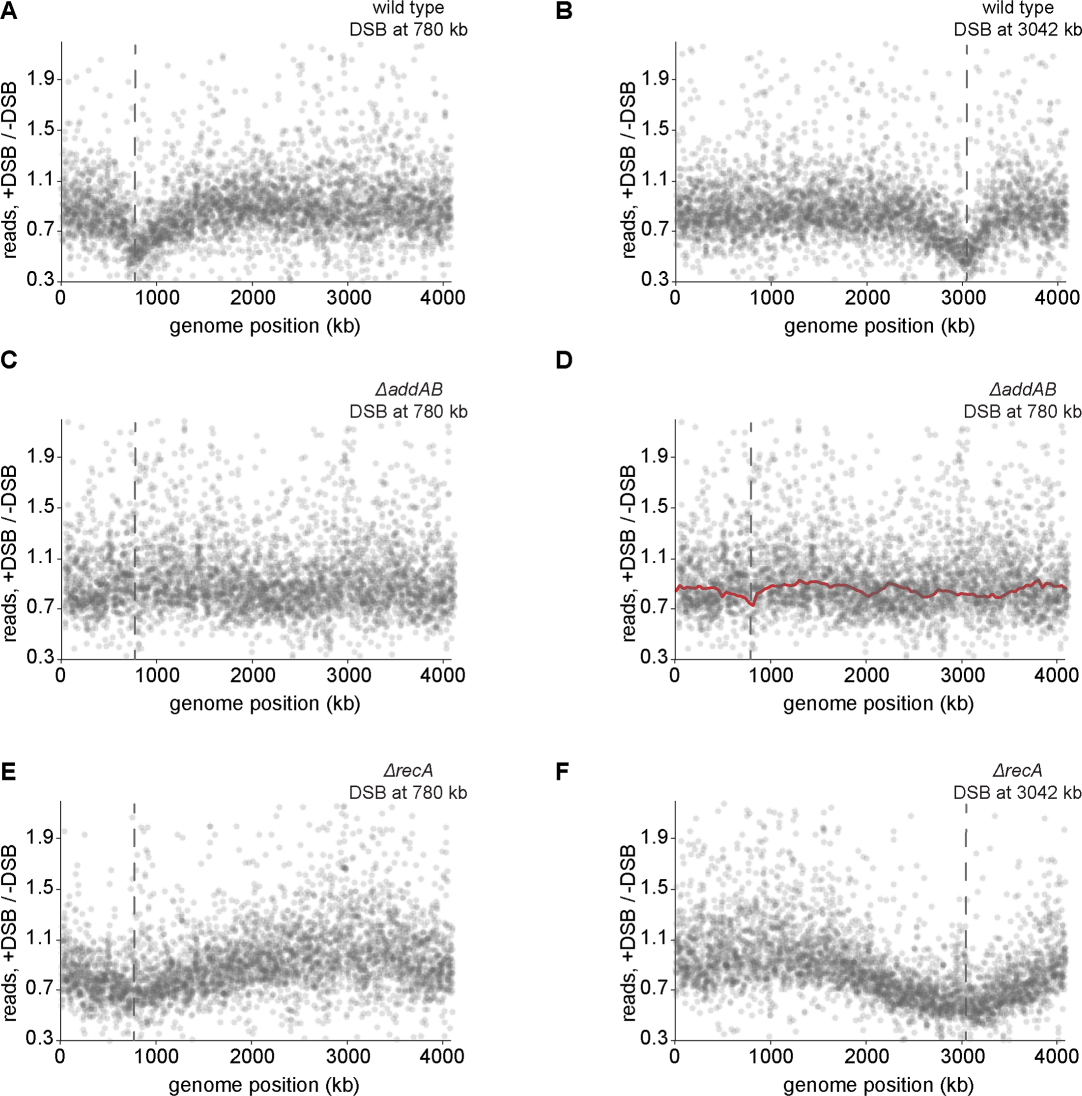
DSB processing and degradation affects transcription of genes near a break site. (A) RNA-seq profile of cells when a DSB is induced +780 kb from the origin for 1 h. Fold difference between DSB induced cells and control (swarmer cells with no DSB induction) is plotted. Each grey dot represents individual transcript levels. Dashed line indicates location of the DSB site. In all cases, average of two independent repeats is plotted. (B) As (A) for a DSB induced at +3042 kb from the origin. (C) RNA-seq profile of cells lacking AddAB when a DSB is induced at +780 kb from the origin. Fold difference between DSB induced cells and control (swarmer cells with no DSB induction) is plotted. Each grey dot represents individual transcript levels. Dashed line indicated location of the DSB site. (D) As in (C) with the DSB processing profile overlaid in red. (E) As in (A) for cells lacking RecA. (F) As in (B) for cells lacking RecA.

Taken together, our results indicate that DSB ends are often subject to extensive degradation and processing, as the AddAB complex may not slowdown at the first *chi* site encountered or may slowdown but continue degrading one strand of the DNA. Thus, AddAB-dependent processing of DSB ends could affect the transcription of genes flanking a break site, as shown recently in yeast [54]. To test this possibility in *Caulobacter*, we performed RNA-seq on swarmer cells subjected to a single DSB. We compared the expression levels of individual genes to untreated swarmer cells. A set of genes associated with the DNA damage response in *Caulobacter* that are found throughout the chromosome increased significantly following a DSB. In addition, we observed a clear decrease in the RNA levels of genes nearest the DSB site (Fig 5A and 5B). These transcriptional profiles correlated well with the DNA processing profiles seen after inducing a DSB. The asymmetry observed in the DNA profiles was also observed in the transcriptional profiles, with larger decreases in transcription on the arm with fewer *chi* sequences. The loss of transcription near a DSB was dependent on AddAB (Fig 5C and 5D).

Because RecA associates with DNA around the break site we also conducted RNA-seq experiments in cells lacking RecA to test the effect of RecA on global and local transcriptional changes. In contrast to the wild type, the decrease in transcription around the break site in Δ*recA* cells was no longer asymmetric and was more extensive compared to *recA*^*+*^ cells (Fig 5E–5F). This result suggests that by slowing AddAB-dependent processing of DSB ends, RecA may help prevent excessive and potentially deleterious losses in transcription.

## Discussion

Homologous recombination in bacteria has been extensively studied *in vitro* and deep mechanistic insights into the function of various protein complexes that participate in the process have been gained using ensemble biochemistry experiments as well as single molecule and structural studies [4,6,8,14,15,17,55]. Recent advances in imaging and sequencing now provide a way to also probe and dissect these mechanisms in the context of living cells and in the context of individual DSBs. Homologous recombination is likely to be profoundly influenced *in vivo* by the structure and organization of the chromosome, and by other concomitant cellular processes such as DNA replication and transcription.

Prior efforts to examine the activities of RecBCD [25,26,31,32,56,57] and AddAB [27–29] *in vivo* have often relied on UV irradiation to create DSBs, but UV light can introduce a range of different types of lesions in the DNA and likely creates a large number of lesions simultaneously. Even in the case of limited UV doses that create only 1–2 lesions per chromosome, the location and timing of the lesions cannot be precisely determined. While the development of I-SceI-based cleavage offers an ability to precisely control the number and timing of DSBs *in vivo* [32,34,37], previous assays for monitoring DNA processing have relied on techniques such as radiolabeled nucleotide incorporation, which has limited resolution, or Southern blotting, which cannot query DNA processing on a global level [31]. Here, we developed a novel *in vivo* assay for monitoring DSB processing by the helicase-nuclease AddAB with relatively high resolution and at a genomic level.

Using this assay, we found that AddAB bidirectionally processes a DSB in an asymmetric manner, with one arm of the chromosome undergoing more degradation than the other arm. This asymmetry correlated with the asymmetric distribution of putative *chi* sequences on the leading and lagging strands, and ectopically inserting these putative *chi* sites was sufficient to significantly slow AddAB-dependent processing (Fig 2). Notably, the difference between the degradation profiles for cells with and without the *chi* sites inserted was detected within ~2–5 kb of the site of insertion (Fig 2F and 2G), demonstrating that our assay has nearly kb resolution and is thus a powerful method for probing DSB processing and repair processes.

Our mathematical modeling suggested that *chi* sites are recognized by and trigger a slowing of AddAB with a probability of 0.23. However, the insertion of an array of 15 sites did not completely stop DNA processing beyond the site of insertion, possibly because clustered *chi* sites are not recognized independently, as noted previously for *chi* recognition by RecBCD in *E*. *coli* [36]. Regardless, our results demonstrate that the putative *chi* site used in these arrays has a demonstrable effect on AddAB-dependent processing of DSBs in *Caulobacter*. Whether these putative *chi* sites are also the sites of increased recombination events as with *chi* sites in *E*. *coli* remains to be determined.

The asymmetry of AddAB-dependent degradation likely leads to an asymmetry in RecA loading, as seen in recent *E*. *coli* RecA ChIP-Seq studies [36]. The loading of RecA onto single-stranded regions of processed DNA ends triggers a significant decrease in AddAB resection or degradation rates as cells lacking *recA* showed more extensive DNA degradation and processing with no obvious asymmetry (Fig 3). These results are consistent with previous work showing that RecA is required to prevent excessive, or 'reckless', DNA degradation by RecBCD in *E*. *coli* [25,26,31,56,58,59], suggesting a conserved mechanism for the regulation of DSB resection in bacteria. Our bacterial two-hybrid results suggest that RecA may regulate AddA through a direct interaction, with AddA probably recruiting RecA to a DSB, as RecBCD does in *E*. *coli* [20,21]. The regulation of AddAB activity likely depends on formation of a RecA filament on ssDNA produced by AddAB, but does not require the SOS response and an increase in RecA levels, nor does it appear to require RecA recombinase activity (Fig 3).

Collectively, our results favor a model in which AddAB activity is effectively self-limiting. In this model, AddAB generates ssDNA upon *chi* recognition, on which RecA can form a filament. AddA may recruit RecA to these regions of ssDNA or somehow promote RecA filament formation, and the subsequent RecA-triggered slowdown in AddAB translocation or nuclease activity [60] limits further resection. This model couples two key events of homologous recombination. The production of RecA-bound DNA, which is competent for homology search, slows or limits additional DSB end processing, enabling homologous recombination to proceed, without any additional impact on the chromosome. The pattern of our wild-type degradation profile (Fig 2B and 2C) suggests that there is active degradation of DNA around a DSB even before *chi* recognition. Our experiments further support the idea that ssDNA is generated after *chi* recognition as the amount of ssDNA we detect via qPCR increased further from the DSB site and as more putative *chi* sites were encountered by AddAB (S3 Fig).

The notion that RecA may directly or indirectly regulate DNA resection by RecBCD in *E*. *coli* has also been suggested previously [25,26,56,58], but precisely how this occurs has been unclear. There are two general models, both of which would lead to "reckless" DNA degradation in the absence of RecA. In one model, as suggested for RecBCD in *E*. *coli* [61–66], RecA loaded onto the ssDNA initially produced by AddAB prevents the reloading of any AddAB or any other exonuclease. In an alternative model, RecA filaments or bundles [48,67] physically limit or slow translocation or DNA resection by AddAB after it recognizes a *chi* site. These models are not mutually exclusive and it is possible that a combination of both occur in the cell. Using a mathematical model to fit our experimentally determined degradation profiles we found two sets of parameters that fit the degradation profiles of wild-type cells. In one, AddAB slowed down only slightly after *chi* recognition, favoring a model in which RecA primarily blocks reassociation of AddAB that has dissociated from the DNA. In the other model, AddAB slowed down more significantly after *chi* recognition, favoring a model in which RecA primarily attenuates AddAB-dependent DNA processing. Direct assessments of the degradation rates *in vivo* using single-cell fluorescence microscopy (Fig 4E–4H) support the latter model in which degradation slows ~4-fold after *chi* recognition, leading us to favor a model in which RecA primarily regulates AddAB translocation. While AddAB may also be influenced by later steps of homologous recombination and repair that were not captured in our experimental set-up, our data indicated that recombination *per se* is not essential for an asymmetric degradation profile in the presence of RecA (Fig 3H and 3I). Whatever the case, limiting AddAB mediated DNA resection is likely important to prevent the excessive loss of DNA if the later steps of homologous recombination are delayed.

As already noted, we also estimated degradation rates in individual cells by tracking the interval of time between loss of fluorescent markers at two different loci near a DSB (Fig 4E–4H). These measurements supported a model in which *chi* recognition by AddAB and the subsequent loading of RecA onto ssDNA slows AddAB-dependent degradation to ~100 bp/s on average. Notably however, the degradation rates measured in individual cells showed much greater variability than was captured in our model (Fig 4). This variability could reflect noise in our measurements of degradation in single cells. Alternatively, it could reflect inherent stochasticity in the degradation rate, or another step of DSB processing, such as the recognition of *chi* sites. Such variability may also partly explain why the degradation profiles show a gradual rather than step-like increase outward from the site of a DSB, although this effect may also arise from variability in when a DSB occurs in individual cells expressing I-SceI.

In sum, our study helps to reveal how AddAB, *chi* sites, and RecA combine to facilitate homologous recombination and maintain genome integrity. In particular, our results highlight an important additional role for RecA. In addition to promoting the pairing of homologous chromosomes, RecA helps to limit DNA resection by AddAB, which, in turn, limits the loss of genetic material and potentially deleterious decreases in gene expression (Fig 5). Given the highly conserved nature of homologous recombination, we speculate that RecA homologs, Rad51 proteins, in eukaryotes may play a similar role.

## Materials and methods

Strains and plasmids used are listed in S1 Table and strain construction details are provided in the supporting information document (S1 Text). Cultures of *Caulobacter* were grown at 30^°^C in PYE and supplemented with antibiotics, as necessary, at appropriate concentrations. For induction of P_*lac*_*-dnaA*, IPTG was added to a final concentration of 0.5 mM. For induction of a DSB, cells were supplemented with 500 μM vanillate unless otherwise indicated. For DSB induction in non-replicating *Caulobacter*, cells were depleted of DnaA for 1.5 h before Percoll density gradient centrifugation to isolate the G1 arrested cells. Swarmer cells were then shifted to conditions (without IPTG) where DnaA is not expressed, and DSBs were induced for indicated times by the addition of 500 μM vanillate. Note that the I-SceI construct used harbors an *ssrA* tag at the C-terminus which likely renders it unstable [68]; this allows the assessment of CFUs at various time points after induction (see S1H Fig) by preventing the accumulation of I-SceI if cells are washed and plated on non-inducing conditions. The wild-type *ssrA* tag is recognized by the ClpXP protease, which then degrades the I-SceI enzyme.

### Bacterial two-hybrid system

Bacterial two hybrid experiments were performed as described in [69]. Briefly, genes of interest were fused to the 5’ or 3’ end of the T18 or T25 fragments in the pUT or pKT vectors [47]. The fusion plasmids were co-transformed into *E*. *coli* BTH101. Co-transformants were grown until saturation in M63 media with maltose, IPTG and appropriate antibiotics and 5 μL of culture was spotted on MacConkey agar (40 g/L) plates with maltose, IPTG and appropriate antibiotics. Plates were incubated at 30^°^C for 2–3 days.

### Western blotting

Cells were pelleted and then resuspended in 1xSDS sample buffer and heated to 95°C for 5 min. Equal amounts of total protein were run on 10% Tris-HCl gels (Bio-Rad) at 150V for separation. Resolved proteins were transferred to polyvinylidene fluoride membranes and probed with 1:5000 dilution of primary antibodies against RecA (Sigma) and secondary horseradish-peroxidase-conjugated antibody (1:5000). Blots were visualized using a FluorChem M imager (ProteinSimple).

### Genomic DNA isolation

*Caulobacter* cells were depleted of DnaA for 1.5 h and G1-arrested cells were then isolated by Percoll density gradient centrifugation. Swarmer cells were then released into DnaA depleting conditions (without IPTG) and DSBs were induced for 1 h by the addition of 500 μM vanillate. Cells were pelleted and genomic DNA was isolated using the DNeasy Blood and Tissue kit from Qiagen. For Mung bean treatment, isolated genomic DNA was incubated at 30°C for 15 min with Mung bean nuclease [70] (NEB) and the DNA was purified with a phenol-chloroform extraction. DNA was sent for whole-genome Illumina sequencing (BioMicroCenter, MIT).

### RNA isolation

*Caulobacter* cells were depleted of DnaA for 1.5 h and G1-arrested cells were then isolated by Percoll density gradient centrifugation. Swarmer cells were then released into DnaA depleting conditions (without IPTG) and DSBs were induced for 1 h by the addition of 500 μM vanillate. Cells were pelleted and frozen in liquid nitrogen for RNA extraction. Cells (in pellets) were lysed by treatment with 400 μL of 65^°^C-preheated Trizol (Thermoscientific) for 10 min on a thermomixer at 200 rpm. They were frozen at -80^°^C for 30 min and then centrifuged at 4^°^C at maximum speed for 5 min. Supernatant was aspirated and added directly to 400 μL of 100% ethanol. The mixture was applied to an RNA-extraction spin column (Zymo Research). The column was then spun at 10000 rpm for 30 s and the spin column was washed with 400 μL of RNA Prewash solution twice and finally with 700 μL of RNA Wash buffer. Residual RNA Wash buffer was removed by an additional centrifugation step. RNA was eluted out with 90 μL of DEPC-treated water. DNase I treatment was carried out to remove any genomic DNA and the RNA was purified using acidic phenol-chloroform extraction. The integrity of the RNA was checked via agarose gel and submitted for Illumina sequencing (BioMicroCenter, MIT).

### DNA sequencing analysis

For analysis of DNA sequencing data, Hiseq 2500 Illumina short reads (40 bp) were mapped to the *Caulobacter* NA1000 reference genome (4.01 Mbp) (NCBI Reference Sequence: NC*-*011916.1) using Bowtie 1 [71] using the following command:

bowtie -m 1 -n 1 --best --strata -p 4 --chunkmbs 512 NA1000*-*2014*-*bowtie --sam *.fastq

The *Caulobacter* NA1000 genome was divided into 4000 bins and mapped Illumina reads were allocated to their corresponding bins to quantify the number of reads in each genomic bin. For samples where the DSB was introduced at 780 kb, datasets were normalized so that the experimental and the control dataset have the same number of Illumina reads between genomic position 2800 kb and 3400 kb. This genomic region was chosen since it is far from the DSB and was not affected by AddAB-induced DNA degradation. For samples where DSB was introduced at 3042 kb, datasets were normalized to have the same number of Illumina reads between genomic position 600 kb and 1200 kb instead. The enrichment between experimental datasets (DSBs were induced) and control dataset (no DSBs) is represented as the ratio of read counts in each bin between the experiment and the control, smoothed using the Lowess function in R with the smoothing bandwidth set to 0.01, and plotted against the genomic positions.

### RNA-seq

For analysis of RNA-seq data, Hiseq 2500 Illumina short reads (40 bp) were mapped back to the *Caulobacter* NA1000 reference genome (NCBI Reference Sequence: NC*-*011916.1) using Bowtie 1 using the following command:

bowtie -m 1 -n 1 --best --strata -p 4 --chunkmbs 512 NA1000*-*2014*-*bowtie --sam *.fastq

The sequencing coverage was computed using BEDTools [72]. The general feature format (gff) file for *Caulobacter* NA1000 was downloaded from NCBI (ftp://ftp.ncbi.nih.gov/genomes/archive/old_genbank/Bacteria/Caulobacter_crescentus_NA1000_uid32027/). The normalized value of reads per kb per million mapped reads (RPKPM) was calculated for each gene by a custom R script to enable comparison of gene expression within and between RNA-seq datasets. The enrichment between experimental datasets (DSBs were induced) and control dataset (no DSBs) is represented as the ratio of RPKPM of each gene between the experiment and the control and plotted against the genomic positions.

### qPCR to estimate rates for DSB formation and total DNA around DSB sites

To estimate rates of DSB formation, qPCR was performed using one set of probes across the DSB site on chromosomal DNA isolated from swarmer or asynchronous Δ*addAB* cells treated with 2 or 500 μM vanillate to induce a DSB +780 kb from the origin. This was normalized to qPCR using probes across a control region (*rpoA*: +1,444 kb) where no DSB is induced. As a control, qPCR across the DSB site was performed on chromosomal DNA isolated from swarmer or asynchronous cells with no DSB induction. Samples were taken 0, 5, 10, 15, 30, 60, 120 and 240 min after DSB induction. The rate of DSB induction was estimated by calculating the slope of the curve (excluding the 240 min time point). To measure total DNA at a DSB and at flanking loci, we performed qPCR using primer pairs as indicated in Fig 1G. The qPCR value measured for each locus following DSB induction was normalized to the distal, control locus, *rpoA*. These normalized values were then divided by similarly normalized values, but from cells in which a DSB was not induced. The resulting ratios (+DSB / -DSB) for each locus were are reported in Fig 1G. The calculations of all qPCR values was done by first generating a standard curve for each oligo pair, with 3-fold dilutions of genomic DNA (and three technical repeats). The average of the 3 technical repeats was then used to calculate the slope and intercept of the curve. The oligo pair was then used for the qPCR measurement described above, using genomic DNA extracted from the following experimental samples: -DSB, +DSB (wild type) and +DSB (Δ*recA*) (3 technical repeats for each, with results averaged). The C_t_ values were converted to amounts using the following formula: 2^((average ct value for sample - intercept)/slope).

### Microscopy assay to estimate rates of degradation between two loci

Fluorescence microscopy was performed on the Zeiss observer Z1 microscope with the LED-Collibri illumination system, 100x oil-immersion objective, Zeiss Temp module to maintain temperature at 30^°^C and a definite focus system for automatic maintenance of focus. Images were acquired via the metamorph imaging system and data analyzed on ImageJ. Swarmer cells were isolated as described above and then grown on PYE + 1.5% low-melting agarose pads with xylose and vanillate and imaged in a glass-bottomed petri dish. Images were acquired every 4 min for ML2402 and every 8 min for ML2401. Rate of degradation was calculated as the number of frames it took to go from the loss of the MipZ marker until the loss of the ParB^pMT1^ marker. Scale bars in figure = 1 μm.

### Data availability

Sequencing data are available in GEO, GSE86913.

## Supporting information

S1 Fig A deep sequencing-based assay for measuring DNA processing after a double-strand break. (A) Flow cytometry profiles of swarmer cells before and after treatment with 500 μM vanillate for 1 h. (B) Representative DSB processing profiles for DSBs induced at +780 kb for 0.5 h (grey) or 1 h (black). 1 h profile is overlaid from Fig 1B. (C) Representative profile for a DSB induced at +780 kb for 1 h. Normalized data is shown in grey, with a Lowess smoothed curve overlaid in black. Location of the DSB site is indicated with a dashed line. (D) As in (C) for a DSB induced at +3042 kb. (E) Comparison of biological replicates when a DSB is induced at +780 kb or +3042 kb. ±300 kb around the break site is compared. (F) Representative processing profile for DSB induced at +780 kb when genomic DNA is treated with Mung Bean nuclease prior to deep sequencing (red). As a control, the profile resulting from genomic DNA not treated with the nuclease prior to sequencing is shown in black (from Fig 1B). (G) Flow cytometry profiles of replicating cells before and after treatment with 2 μM or 500 μM vanillate for 1 h. (H) Fold change in Colony Forming Units (CFU) upon DSB induction at +780 kb in swarmer cells. Samples were plated prior to DSB induction (0) and 10, 20, 40, 60 and 120 min after the addition of vanillate. Error bars represent standard deviation between two independent repeats.
(TIF)

S2 Fig DNA degradation around a double-strand break is asymmetric due to *chi* sites. (A-B) Genome-wide profiles from Fig 2B and 2C are shown. Positions of *chi* sequences on the leading strand (A) or lagging strand (B) with respect to a DSB site at +780 kb are overlaid on the profile. (C-D) Positions of *chi* sequences on the leading strand (C) or lagging strand (D) with respect to a DSB site at +3042 kb are overlaid on the DSB processing profile. (E-F) Genome-wide profiles from Fig 2E and 2F are shown. A repeat of 15 *chi* sequences (*chi*^*for*^) was inserted +30 kb (E) or +100 kb (F) from the DSB site at +780 kb and the profile is shown in green. As a control, the *chi* orientation is flipped (*chi*^*rev*^) at the same location and the profile is shown in orange. Degradation profile of a DSB induced at +780 kb is shown from Fig 1B (black).
(TIF)

S3 Fig Effect of *chi* sites on AddAB is dependent on RecA. (A-B) qPCR was performed using the primer pairs at the genomic positions indicated in Δ*recA* cells. The location of putative *chi* sites on the top and bottom strands are indicated with red and blue, respectively, tick marks (also see Fig 2A). Samples were collected before (0 min) and 60 min after DSB induction. qPCR at a distal, unprocessed control site (*rpoA*: +1,444 kb) was also performed. qPCR values at each locus normalized to *rpoA* are plotted for two independent repeats (a and b). DSB processing profile values for the same loci are also shown. (C) Representative DSB processing profile of *ΔrecA* swarmer cells when a DSB is induced at +780 kb for 1 h. Normalized data are shown in pink, with a Lowess smoothed curve overlaid in red. Location of the DSB site is indicated with a dashed line. Profile of wild type swarmer cells from S1A Fig is also shown. (D) As in panel A, but for a DSB induced at +3042 kb. (E, F) Bacterial-two-hybrid assay on a single plate, showing all interactions along with controls. (G) Bacterial-two-hybrid assay and Western blot showing that RecA(K83A) used in Fig 3D is not deficient in interaction with AddA or expression inside the cell. Western blot for RecA(N304D) is also shown. (H) RecA(N304D) is sensitive to low levels of DSB induction (2 μM vanillate), comparable to *ΔrecA* cells. Each spot is a 10-fold dilution.
(TIF)

S4 Fig Estimation of rates of DSB formation. (A) Histogram showing distribution of number of DSB events per cell in 100 s intervals expected over time (from experimental results) from the time of I-SceI induction to DSB onset. Dashed line indicates the value 1 h after DSB induction (B) Graph showing fraction of cells expected to have DSB over time from the time of I-SceI induction to DSB onset. Dashed line indicates the value 1 h after DSB induction. See supporting information for details. (C) Rates of DSB formation were estimated with qPCR using probes across the DSB site in synchronous (swarmer) and asynchronous populations of cells treated with 2 and 500 μM vanillate. Samples were collected before (0 min) and 5, 10, 15, 30, 60, 120 and 240 min after DSB induction. Results were normalized to qPCR across a control site (*rpoA*). Error bars represent SD between two biological replicates. (D) Rates of DSB formation were calculated from panel C.
(TIF)

S5 Fig AddAB-dependent DNA resection is attenuated after *chi* recognition. (A) Simulation profiles obtained for a model where post-*chi* AddAB degradation rates are estimated. Pre-*chi* AddAB degradation rate is 400 bp/s and *chi* recognition probability is found to be ~0.22. DSB is induced at +3042 kb for 1, 2 or 4 h. Experimental profiles are overlaid in black, grey and brown respectively. Simulation profiles are in green (B-C) Simulation profiles obtained for Model 1 (red) and 2 (blue) that best fit the *in vivo* DNA processing profiles for a DSB induced at +3042 kb for 1 h and 2 h samples respectively. Experimental profiles from Fig 1 are overlaid in black and grey. (D) Profile for a DSB induced at +3042 kb for 4 h is shown in brown. Simulation profiles (for the 4h time point) predicted for Model 1 (red) and Model 2 (blue) are overlaid.
(TIF)

S6 Fig Root-mean-squared deviation (RMSD) fits for simulated models. (A) Graph showing RMS deviation for *chi* recognition probability (p_*chi*_) for models considered to best fit the *in vivo* wild-type DSB processing profiles. In all cases, dashed red lines represent the best fits and 95% confidence intervals. (B) Graph showing RMS deviation as a function of post-*chi* AddAB degradation rate (k_slow_) for Model 2. Post-*chi* AddAB degradation rate (k_slow_) for Model 1 is fixed at 340 bp/s. (C-D) Graphs showing RMS deviations as a function of post-*chi* AddAB dissociation rate (k_off_) for Models 1 and 2.
(TIF)

S1 Table Strains, plasmids and primers used in this study. (PDF)

S2 Table qPCR data and locations of *chi* sites near DSB site introduced at +780 kb. (XLSX)

S1 Text Supporting methods and results. (DOCX)
