# Supplementary figures and images for: Global analysis of double-strand break processing reveals in vivo properties of the helicase-nuclease complex AddAB
Source: PLoS Genet. 2017 May 10;13(5):e1006783. doi: 10.1371/journal.pgen.1006783 (PMC5443536; doi:10.1371/journal.pgen.1006783)

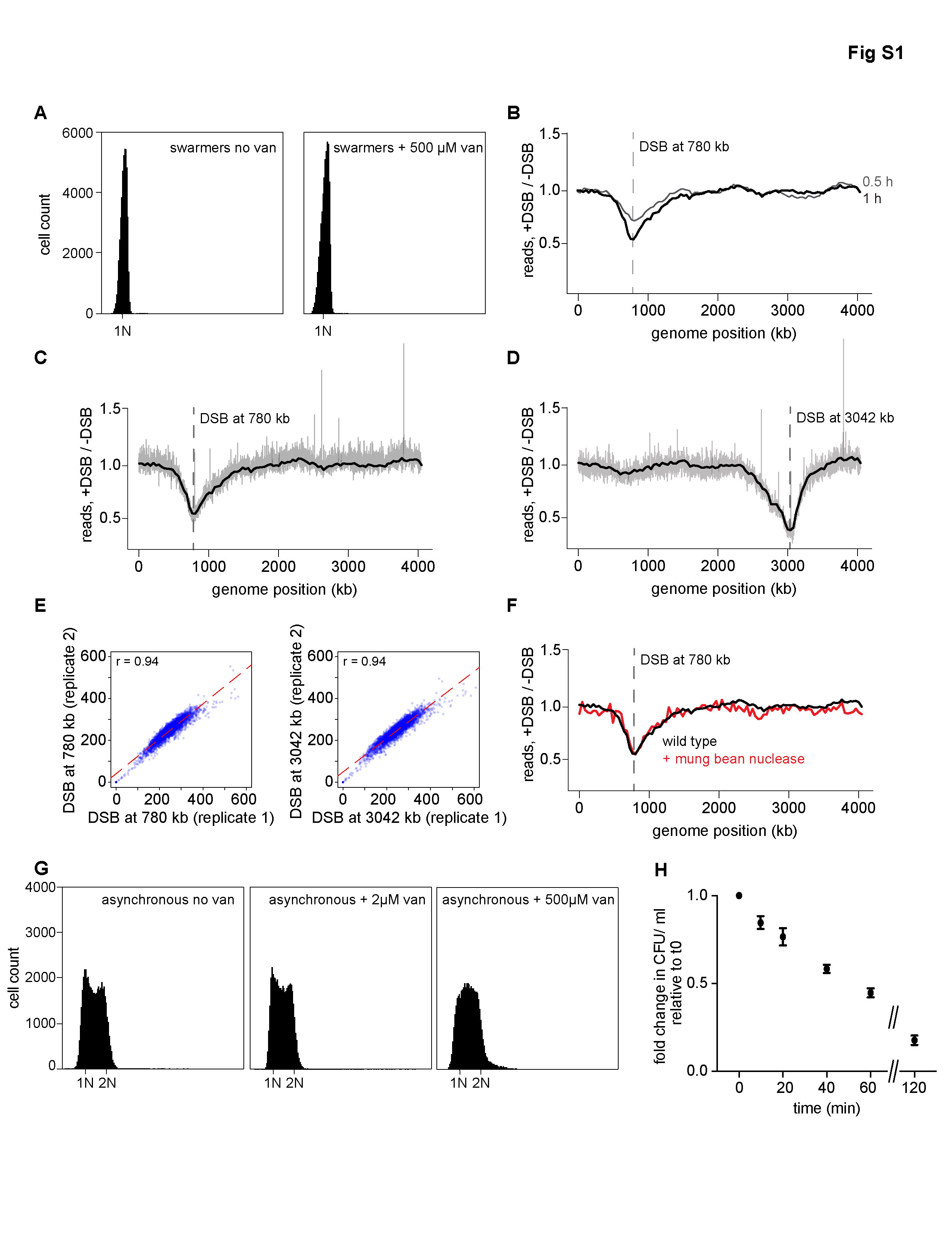

Supplement: S1 Fig — (A) Flow cytometry profiles of swarmer cells before and after treatment with 500 μM vanillate for 1 h. (B) Representative DSB processing profiles for DSBs induced at +780 kb for 0.5 h (grey) or 1 h (black). 1 h profile is overlaid from Fig 1B. (C) Representative profile for a DSB induced at +780 kb for 1 h. Normalized data is shown in grey, with a Lowess smoothed curve overlaid in black. Location of the DSB site is indicated with a dashed line. (D) As in (C) for a DSB induced at +3042 kb. (E) Comparison of biological replicates when a DSB is induced at +780 kb or +3042 kb. ±300 kb around the break site is compared. (F) Representative processing profile for DSB induced at +780 kb when genomic DNA is treated with Mung Bean nuclease prior to deep sequencing (red). As a control, the profile resulting from genomic DNA not treated with the nuclease prior to sequencing is shown in black (from Fig 1B). (G) Flow cytometry profiles of replicating cells before and after treatment with 2 μM or 500 μM vanillate for 1 h. (H) Fold change in Colony Forming Units (CFU) upon DSB induction at +780 kb in swarmer cells. Samples were plated prior to DSB induction (0) and 10, 20, 40, 60 and 120 min after the addition of vanillate. Error bars represent standard deviation between two independent repeats. (TIF) [file pgen.1006783.s001.tif]

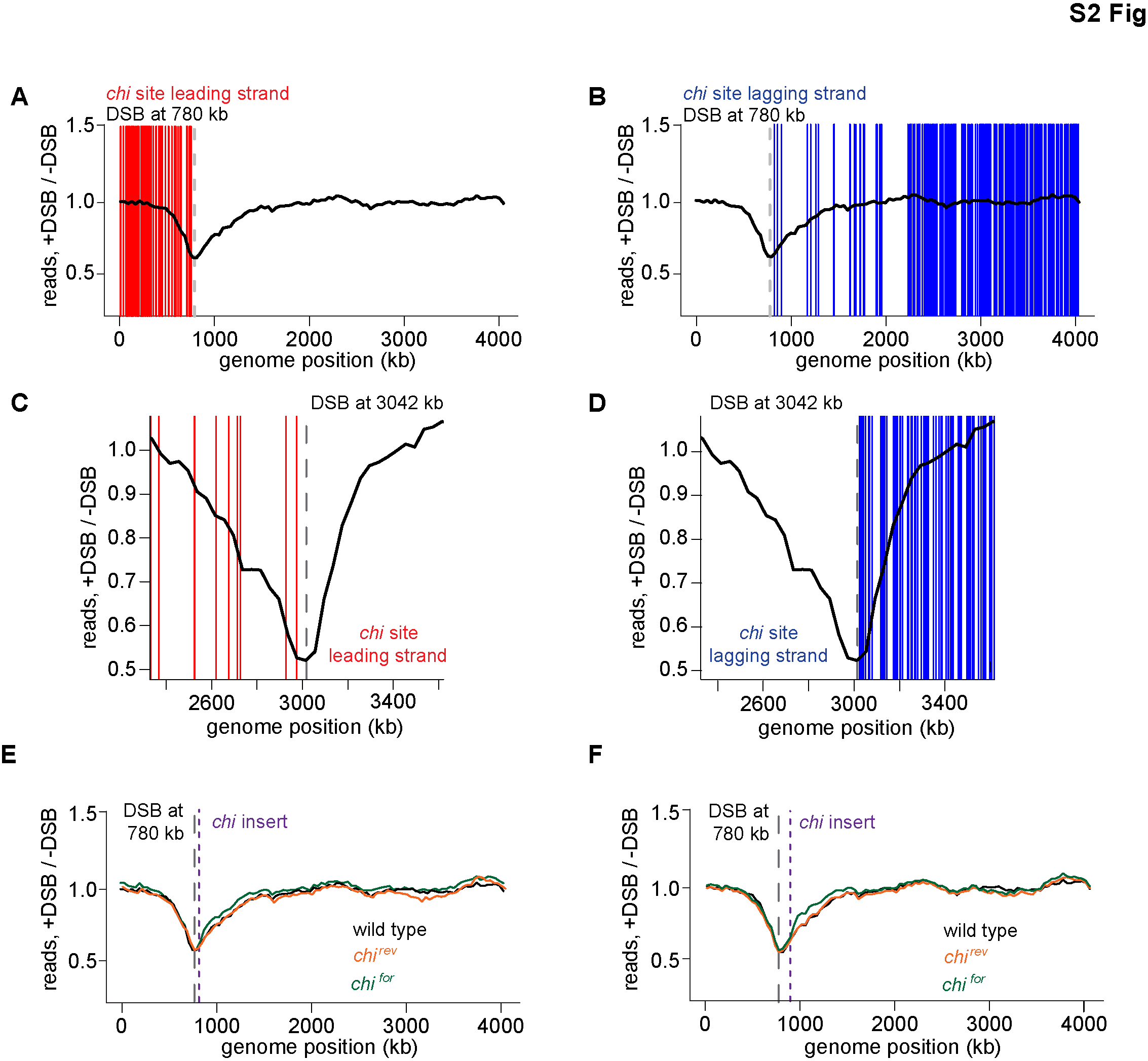

Supplement: S2 Fig — (A-B) Genome-wide profiles from Fig 2B and 2C are shown. Positions of chi sequences on the leading strand (A) or lagging strand (B) with respect to a DSB site at +780 kb are overlaid on the profile. (C-D) Positions of chi sequences on the leading strand (C) or lagging strand (D) with respect to a DSB site at +3042 kb are overlaid on the DSB processing profile. (E-F) Genome-wide profiles from Fig 2E and 2F are shown. A repeat of 15 chi sequences (chifor) was inserted +30 kb (E) or +100 kb (F) from the DSB site at +780 kb and the profile is shown in green. As a control, the chi orientation is flipped (chirev) at the same location and the profile is shown in orange. Degradation profile of a DSB induced at +780 kb is shown from Fig 1B (black). (TIF) [file pgen.1006783.s002.tif]

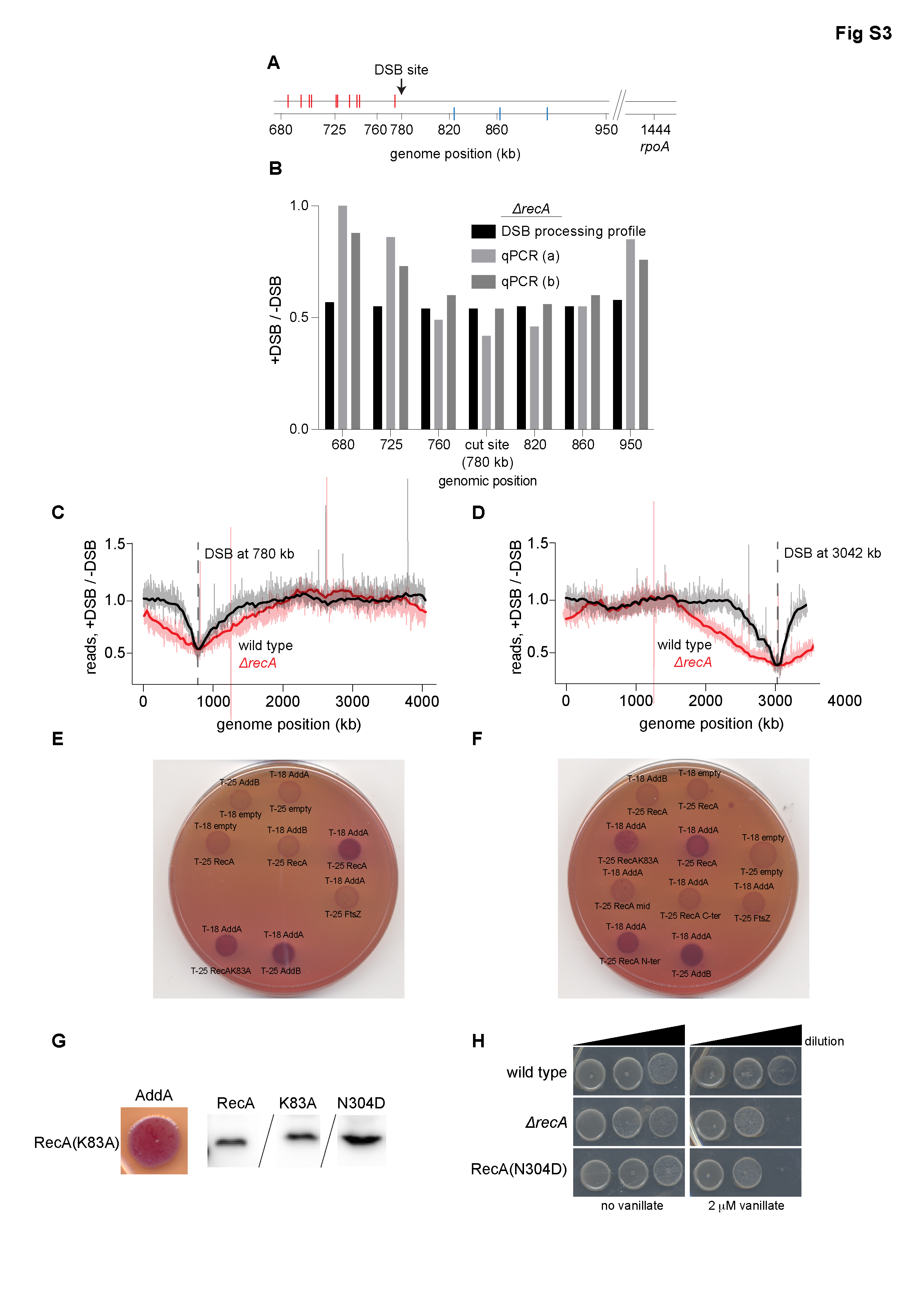

Supplement: S3 Fig — (A-B) qPCR was performed using the primer pairs at the genomic positions indicated in ΔrecA cells. The location of putative chi sites on the top and bottom strands are indicated with red and blue, respectively, tick marks (also see Fig 2A). Samples were collected before (0 min) and 60 min after DSB induction. qPCR at a distal, unprocessed control site (rpoA: +1,444 kb) was also performed. qPCR values at each locus normalized to rpoA are plotted for two independent repeats (a and b). DSB processing profile values for the same loci are also shown. (C) Representative DSB processing profile of ΔrecA swarmer cells when a DSB is induced at +780 kb for 1 h. Normalized data are shown in pink, with a Lowess smoothed curve overlaid in red. Location of the DSB site is indicated with a dashed line. Profile of wild type swarmer cells from S1A Fig is also shown. (D) As in panel A, but for a DSB induced at +3042 kb. (E, F) Bacterial-two-hybrid assay on a single plate, showing all interactions along with controls. (G) Bacterial-two-hybrid assay and Western blot showing that RecA(K83A) used in Fig 3D is not deficient in interaction with AddA or expression inside the cell. Western blot for RecA(N304D) is also shown. (H) RecA(N304D) is sensitive to low levels of DSB induction (2 μM vanillate), comparable to ΔrecA cells. Each spot is a 10-fold dilution. (TIF) [file pgen.1006783.s003.tif]

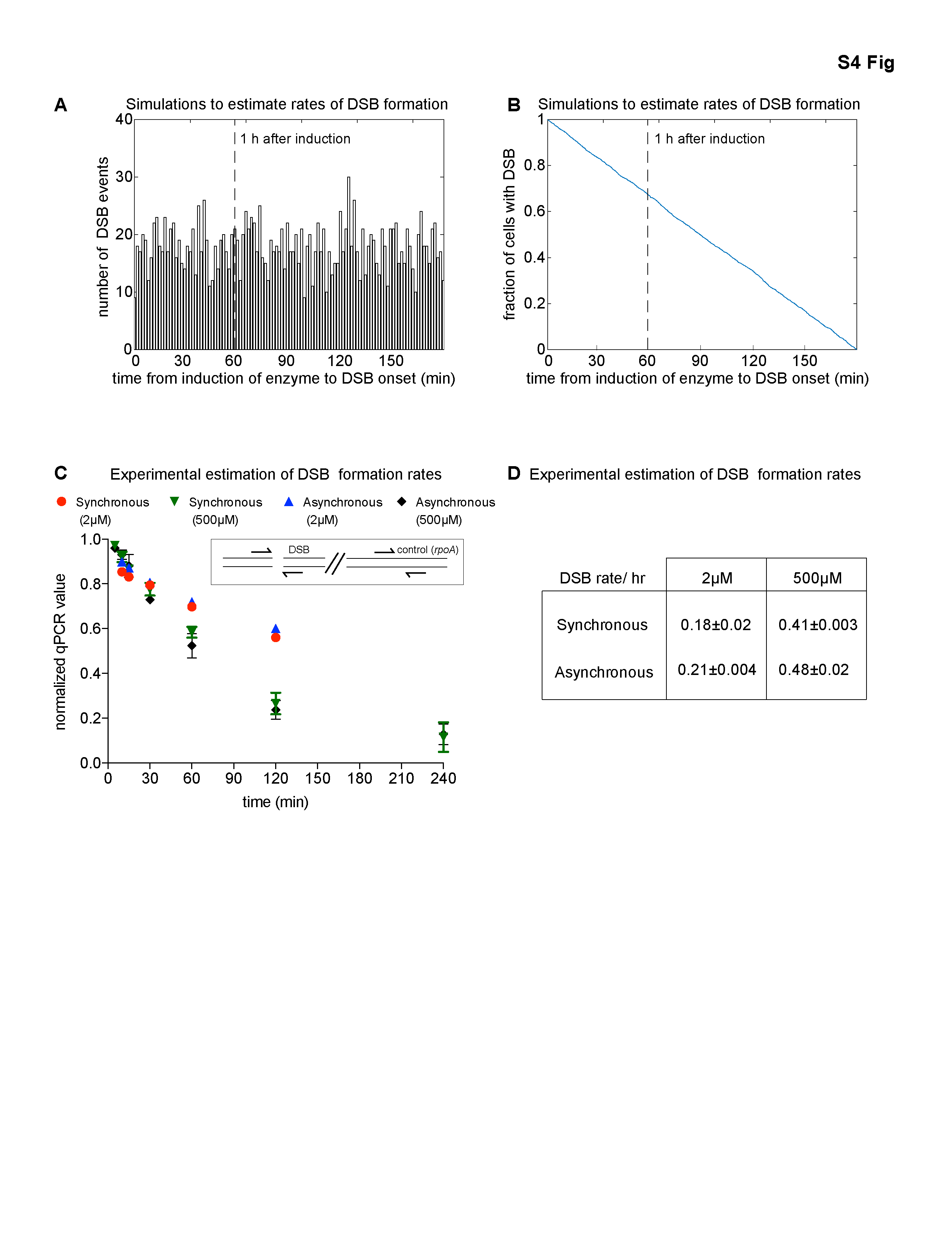

Supplement: S4 Fig — (A) Histogram showing distribution of number of DSB events per cell in 100 s intervals expected over time (from experimental results) from the time of I-SceI induction to DSB onset. Dashed line indicates the value 1 h after DSB induction (B) Graph showing fraction of cells expected to have DSB over time from the time of I-SceI induction to DSB onset. Dashed line indicates the value 1 h after DSB induction. See supporting information for details. (C) Rates of DSB formation were estimated with qPCR using probes across the DSB site in synchronous (swarmer) and asynchronous populations of cells treated with 2 and 500 μM vanillate. Samples were collected before (0 min) and 5, 10, 15, 30, 60, 120 and 240 min after DSB induction. Results were normalized to qPCR across a control site (rpoA). Error bars represent SD between two biological replicates. (D) Rates of DSB formation were calculated from panel C. (TIF) [file pgen.1006783.s004.tif]

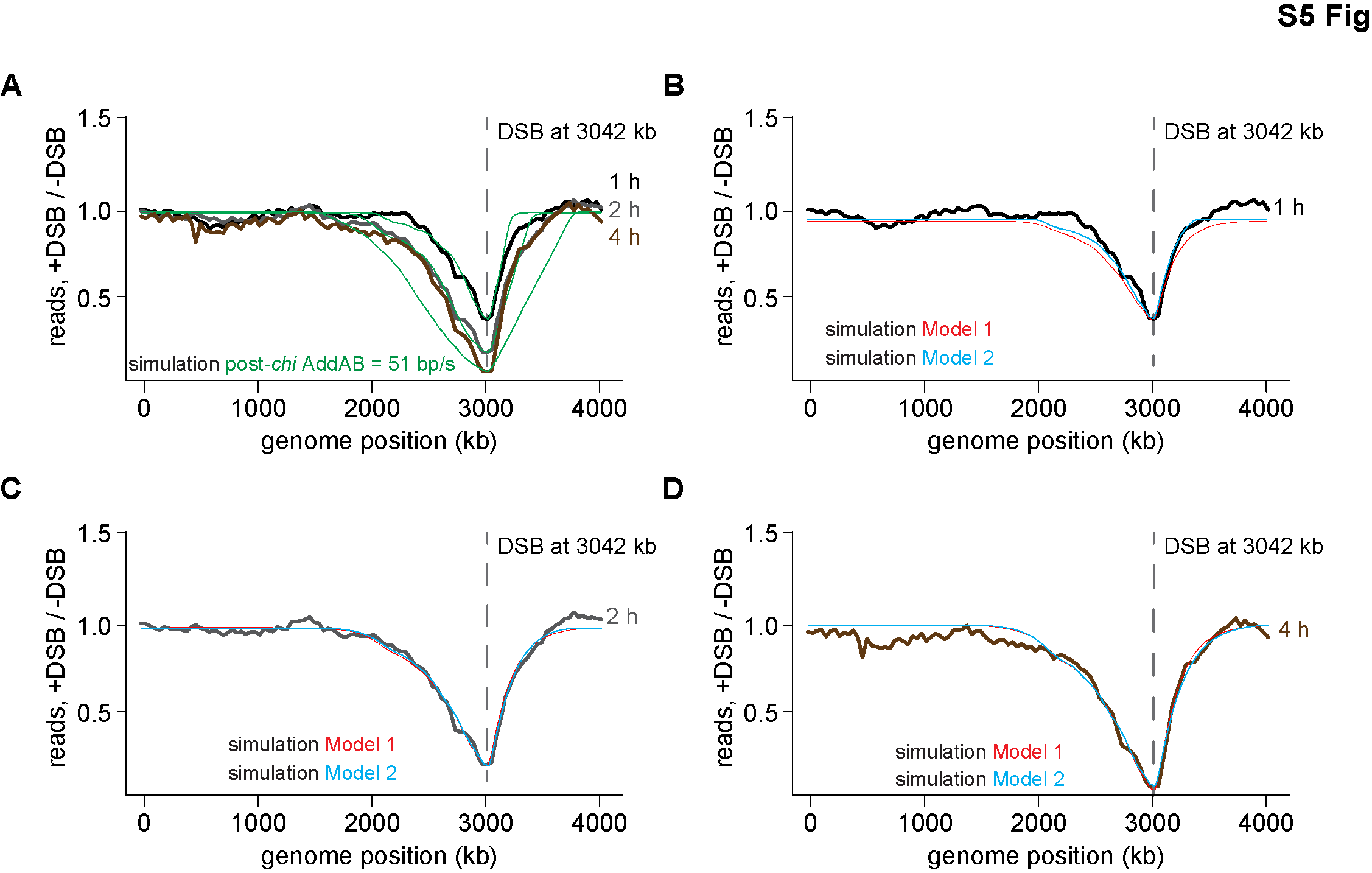

Supplement: S5 Fig — (A) Simulation profiles obtained for a model where post-chi AddAB degradation rates are estimated. Pre-chi AddAB degradation rate is 400 bp/s and chi recognition probability is found to be ~0.22. DSB is induced at +3042 kb for 1, 2 or 4 h. Experimental profiles are overlaid in black, grey and brown respectively. Simulation profiles are in green (B-C) Simulation profiles obtained for Model 1 (red) and 2 (blue) that best fit the in vivo DNA processing profiles for a DSB induced at +3042 kb for 1 h and 2 h samples respectively. Experimental profiles from Fig 1 are overlaid in black and grey. (D) Profile for a DSB induced at +3042 kb for 4 h is shown in brown. Simulation profiles (for the 4h time point) predicted for Model 1 (red) and Model 2 (blue) are overlaid. (TIF) [file pgen.1006783.s005.tif]

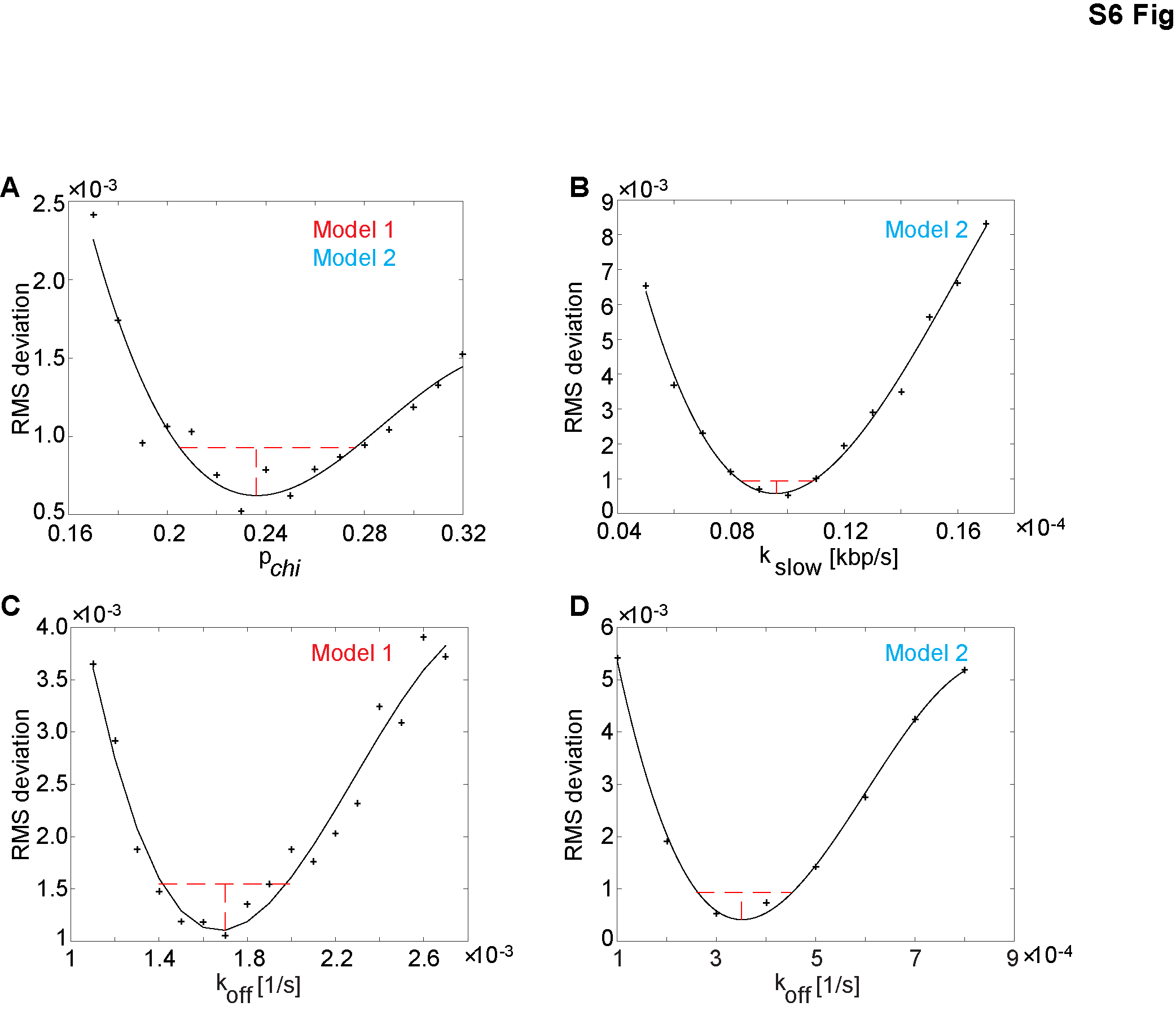

Supplement: S6 Fig — (A) Graph showing RMS deviation for chi recognition probability (pchi) for models considered to best fit the in vivo wild-type DSB processing profiles. In all cases, dashed red lines represent the best fits and 95% confidence intervals. (B) Graph showing RMS deviation as a function of post-chi AddAB degradation rate (kslow) for Model 2. Post-chi AddAB degradation rate (kslow) for Model 1 is fixed at 340 bp/s. (C-D) Graphs showing RMS deviations as a function of post-chi AddAB dissociation rate (koff) for Models 1 and 2. (TIF) [file pgen.1006783.s006.tif]
