# Supplementary material for: Global analysis of double-strand break processing reveals in vivo properties of the helicase-nuclease complex AddAB
Source: PLoS Genet. 2017 May 10;13(5):e1006783. doi: 10.1371/journal.pgen.1006783 (PMC5443536; doi:10.1371/journal.pgen.1006783)
Supplement: S1 Table — (PDF) [file pgen.1006783.s007.pdf]

S1 Table: Strains, plasmids and primers used in this study

Strain	Genotype	Source	Description
ML2464	P_{lacI}-*lacI* (*hfaA* locus); P_{lac}-*dnaA* (*dnaA* locus); I-SceI site (after CCNA_00727)::*tet*^{R}; *parS*^{pMT1} site (after CCNA_00747)::*spec*^{R}; P_{van}-*I-SceI*::*chlor*^{R};	[1]	Used to introduce DSB +780 kb from the origin
ML2465	P_{lacI}-*lacI* (*hfaA* locus); P_{lac}-*dnaA* (*dnaA* locus); I-SceI site (after CCNA_03821)::*tet*^{R}; *parS*^{pMT1} site (after CCNA_03776)::*spec*^{R}; P_{van}-*I-SceI*::*chlor*^{R};	This study	Used to introduce DSB +3042 kb from the origin
ML2466	P_{lacI}-*lacI* (*hfaA* locus); P_{lac}-*dnaA* (*dnaA* locus); I-SceI site (after CCNA_00727)::*tet*^{R}; *parS*^{pMT1} site (after CCNA_00747)::*spec*^{R}; P_{van}-*I-SceI*::*chlor*^{R}; *ΔaddAB*::*gent*^{R}	This study	Strain with cut site at +780 kb, lacking *addAB*. Figure 1D.
ML2467	P_{lacI}-*lacI* (*hfaA* locus); P_{lac}-*dnaA* (*dnaA* locus); I-SceI site (after CCNA_00727)::*tet*^{R}; *parS*^{pMT1} site (after CCNA_00747)::*spec*^{R}; P_{van}-*I-SceI*::*chlor*^{R}; *chi*^{For} (after CCNA_00774)::*gent*^{R}	This study	Strain with cut site at +780 kb, with *chi* insertion. Figures 2, S2.
ML2468	P_{lacI}-*lacI* (*hfaA* locus); P_{lac}-*dnaA* (*dnaA* locus); I-SceI site (after CCNA_00727)::*tet*^{R}; *parS*^{pMT1} site (after CCNA_00747)::*spec*^{R}; P_{van}-*I-SceI*::*chlor*^{R}; *chi*^{Rev} (after CCNA_00774)::*gent*^{R}	This study	Strain with cut site at +780 kb, with *chi* insertion. Figures 2, S2.
ML2469	P_{lacI}-*lacI* (*hfaA* locus); P_{lac}-*dnaA* (*dnaA* locus); I-SceI site (after CCNA_00727)::*tet*^{R}; *parS*^{pMT1} site (after CCNA_00747)::*spec*^{R}; P_{van}-*I-SceI*::*chlor*^{R}; *chi*^{For} (after CCNA_00836)::*gent*^{R}	This study	Strain with cut site at +780 kb, with *chi* insertion. Figures 2, S2.
ML2470	P_{lacI}-*lacI* (*hfaA* locus); P_{lac}-*dnaA* (*dnaA* locus); I-SceI site (after CCNA_00727)::*tet*^{R}; *parS*^{pMT1} site (after CCNA_00747)::*spec*^{R}; P_{van}-*I-SceI*::*chlor*^{R}; *chi*^{Rev} (after CCNA_00836)::*gent*^{R}	This study	Strain with cut site at +780 kb, with *chi* insertion.

			Figures 2, S2.
ML2471	P_{lacI}-*lacI (hfaA* locus); P_{lac}-*dnaA* (*dnaA* locus); I-SceI site (after CCNA_00727)::*tet*^{R}; *parS*^{pMT1} site (after CCNA_00747)::*spec*^{R}; P_{van}-*I-SceI::chlor*^{R}; *ΔrecA*::*gent*^{R}	This study	Strain with cut site at +780 kb, lacking *recA.*
ML2000	P_{lacI}-*lacI (hfaA* locus); P_{lac}-*dnaA* (*dnaA* locus)	[2]	Strain for *dnaA* depletion.
ML2425	P_{lacI}-*lacI (hfaA* locus); P_{lac}-*dnaA* (*dnaA* locus); I-SceI site (after CCNA_03821)::*tet*^{R}; *parS*^{pMT1} site (after CCNA_03776)::*spec*^{R}; P_{van}-*I-SceI::chlor*^{R}; *ΔrecA*::*gent*^{R}	This study	Strain with cut site at +3042 kb, lacking *recA.*
ML2447	P_{lacI}-*lacI (hfaA* locus); P_{lac}-*dnaA* (*dnaA* locus); I-SceI site (after CCNA_00727)::*tet*^{R}; *parS*^{pMT1} site (after CCNA_00747)::*spec*^{R}; P_{van}-*I-SceI::chlor*^{R}; *ΔrecA*::*gent*^{R}; P_{recA}-*recA(K83A)* (*xyl* locus)::*kan*^{R}	This study	Strain with cut site at +780 kb, lacking *recA,* expressing *recA(K83A).* Figure 3, S3.
ML2644	P_{lacI}-*lacI (hfaA* locus); P_{lac}-*dnaA* (*dnaA* locus); I-SceI site (after CCNA_00727)::*tet*^{R}; *parS*^{pMT1} site (after CCNA_00747)::*spec*^{R}; P_{van}-*I-SceI::chlor*^{R}; *lexA**::*kan*^{R}	This study	Strain with cut site at +780 kb, expressing a non-cleavable mutant of *lexA.* Figure 3.
ML2401	P_{lacI}-*lacI (hfaA* locus); P_{lac}-*dnaA* (*dnaA* locus); I-SceI site (after CCNA_00029)::*tet*^{R}; P_{van}*I-SceI::chlor*^{R}; *mipZ-YFP::kan*^{R}; *parS*^{pMT1} site (after CCNA_03678)::*spec*^{R}; P_{xyl}*CFP-parB*^{pMT1}*::gent*^{R}	[1]	Strain with cut site at +30 kb, with markers to visualize the origin and a chromosomal locus -130kb from the break site. Figure 4.
ML2402	P_{lacI}-*lacI (hfaA* locus); P_{lac}-*dnaA* (*dnaA* locus); I-SceI site (after CCNA_00029)::*tet*^{R}; P_{van}-*I-SceI::chlor*^{R}; *mipZ-CFP::gent*^{R}; *parS*^{pMT1} after CCNA_03776)::*spec*^{R}; P_{xyl}-*YFP-parB*^{pMT1}*,mCherry-parB*^{P1}*::kan*^{R}	[1]	Strain with cut site at +30 kb, with markers to visualize the origin and a

			chromosomal locus -230kb from the break site. Figure 4.

Plasmids:

Plasmid	Background	Description	Source
pNPTS138	DH5α	Integration vector (*kan*^{R}) with *sacB* counterselection	[3]
pMT675	DH5α	Vector for integration at P_{van} (*chlor*^{R})	[4]
pMT664	DH5α	Vector for integration of CFP to the c terminus of genes at native locus (*kan*^{R})	[4]
pMT697	DH5α	Vector for integration at P_{xyl} (*kan*^{R})	[4]
pMT635	DH5α	Vector for integration at site of interest (*gent*^{R})	[4]
ML2430	DH5α	pMT675-based plasmid for integration of *I-SceI-ssrA* at P_{van}	[1]
ML2438	DH5α	pNPTS138-based plasmid for insertion of I-SceI site after CCNA_00727	[1]
ML2439	DH5α	pNPTS138-based plasmid replacement of *addAB* with *gent*^{R}	[1]
ML2461	DH5α	pMT635-based plasmid for integration of *gent*^{R} near *ΔrecA* (after CCNA_01139)	[1]
ML2647	DH5α	pNPTS138-based plasmid for insertion of I-SceI site after CCNA_03821	This study

ML2648	DH5α	pMT697-based plasmid for integration of P_{recA}-*recA* at the *xyl* locus	This study
ML2649	DH5α	pMT697-based plasmid for integration of P_{recA}-*recA(K83A)* at the *xyl* locus	This study
ML2650	DH5α	pMT635-based plasmid for integration of *chi*^{For} after CCNA_00774	This study
ML2651	DH5α	pMT635-based plasmid for integration of *chi*^{Rev} after CCNA_00774	This study
ML2652	DH5α	pMT635-based plasmid for integration of *chi*^{For} after CCNA_00836	This study
ML2653	DH5α	pMT635-based plasmid for integration of *chi*^{Rev} after CCNA_00836	This study
ML2654	DH5α	pKT25-based plasmid expressing *recA* for bacterial-two-hybrid assay	This study
ML2655	DH5α	pKT25-based plasmid expressing *recA*^{KA} for bacterial-two-hybrid assay	This study
ML2656	DH5α	pUT18C-based plasmid expressing *addA* for bacterial-two-hybrid assay	This study
ML2657	DH5α	pKT25-based plasmid expressing *addB* for bacterial-two-hybrid assay	This study
ML2658	DH5α	pUT18C-based plasmid expressing *addB* for bacterial-two-hybrid assay	This study
ML2659	DH5α	pUT18C-based plasmid expressing *recA* for bacterial-two-hybrid assay	This study

ML2660	DH5α	pKT25-based plasmid expressing *addA* for bacterial-two-hybrid assay	This study
ML2661	DH5α	pUT18C-based plasmid expressing *ftsZ* for bacterial-two-hybrid assay	[5]

Primers:

Details of oligos used have been provided in the supporting information.

Description	Sequence
I-SceI_forward	cacccatatggaaaacatcaaaaaaaaccaggtaat
I-SceI_reverse	ttatgctagcttacgacgcatccgcgtagttttcagattcttccgcgaagttatcgttatccgctttcaggaaagtttcggaggag
tet_forward	cggtatcgataagcttgatatcgaattcatgaag
tet_reverse	ctgcaggaattcaagaagttcctattctctagaaag
Isce1_ccna03821_up_forward	ttatgaattctcgcgcacgagacctggac
Isce1_ccna003821_up_reverse	gaacccgaccgataggcccgctcaaacacgcggcatggc
Isce1_ccna03821_down_forward	ggcaacgagccgatcgctgatgcgccccctccgtctcg
Isce1_ccna03821_down_reverse	ttatgctagcggccgacaggttgaaggcgt
P-$_{recA}$*recA*_forward	caccgccggacatcagcgcggcgccattcgccatcagtaagcccgcaacccttgca
P-$_{recA}$*recA*_reverse	gtaaggagaaaataccgcatcaggcgccatctagtcctcttcgccctcttccgg

RecA^{KA}_up	ccggaaagctcgggcgcgaccaccctggccctg
RecA^{KA}_down	cagggccagggtggtcgcgcccgagctttccgg
chi_forward	cttcagacttgccggccaggc
chi_reverse	gtaacgttcgaattctccggagctccgcggggtgccagtctacgaa
ccna_00774_forward	ttatcatatggccatgcccgacgacgcttc
ccna_00774_reverse	gcctggccggcaagtctgaagttaagtccgccagcgcagcgtc
ccna_00836_forward	ttatcatatggcgctaaacagcatcaatacgaactc
ccna_00836_reverse	gcctggccggcaagtctgaagctatcggaaaagactgaggatcgccg
B2h_recA_forward	ttatggatcccatgacaagtcaggcggctttgaaact
B2h_recA_reverse	ttatggtaccctagtcctcttcgccctcttccgg
B2h_addA_forward	ttatggatcccatgcacgacccccagcgc
B2h_addA_reverse	ttatggtaccttaagccagccgcgccaga
B2h_addB_forward	ttatggatcccatgagcgggtccgcgcc
B2h_addB_reverse	ttatggtacctcattcgccctctccgtcatcg
rpoA forward	acatcgtctacatcggcgac
rpoA reverse	ggcgagcacttccttgatct
780kb forward	cctggaattccggctcag
780kb reverse	acgttgaaggaccgagaaag

760kb forward	ccaagaacctctccgacaat
760kb reverse	ccggtagccatgtttgtatct
725kb forward	cttccggatcggctagaac
725kb reverse	cggaactcaggctctaaagat
680kb forward	tcgccaaggctcagaatatc
680kb reverse	acaacacattggaagggtagaa
820kb forward	cgatcttcgcgatccactatg
820kb reverse	cgcttcgttgctgggatta
860kb forward	tgatcctcaagaccatcaagc
860kb reverse	attgtgaggcgctcctttag
950kb forward	cgatcctgcgacgcttt
950kb reverse	tcgatcagcgtatcgatgttg
