# Supplementary material for: Global analysis of double-strand break processing reveals in vivo properties of the helicase-nuclease complex AddAB
Source: PLoS Genet. 2017 May 10;13(5):e1006783. doi: 10.1371/journal.pgen.1006783 (PMC5443536; doi:10.1371/journal.pgen.1006783)
Supplement: S1 Text — (DOCX) [file pgen.1006783.s009.docx]

Supporting Methods

Strain construction

Transductions were performed using ΦCR30 [6]. Chromosomal integrations and deletions were made using either a two-step recombination method with a *sacB* counter-selection marker [3] or using vectors described in [4].

For ML2464, first the I-SceI site with *tet* resistance marker was inserted between CCNA_00727 and CCNA_00728 following a two-step recombination protocol using ML2438 transformed into CB15N. A lysate of the resultant strain was used for transduction into ML2000 with *parS*^{pMT1} inserted after CCNA_00747. Separately, ML2430 was transformed into CB15N for insertion of *I-SceI* enzyme at the van locus by single crossover integration. This was transduced into ML2000 with the I-SceI site after CCNA_00727, generating ML2464.

For ML2465, first the I-SceI site with *tet* resistance marker was inserted between CCNA_03821 and CCNA_03822 following a two-step recombination protocol using ML2647 transformed into CB15N. A lysate of the resultant strain was used for transduction into ML2000 with *parS*^{pMT1} inserted after CCNA_03776. Separately, ML2430 was transformed into CB15N for insertion of *I-SceI* enzyme at the van locus by single crossover integration. This was transduced into ML2000 with the I-SceI site after CCNA_03821, generating ML2465.

For ML2466, first ML2439 was transformed into CB15N in order to construct a deletion *addBA,* replacing it with a *gent* resistance cassette, following a two-step recombination protocol. This was transduced into ML2464 to give ML2466.

For ML2467-2470, first ML2650-2653 were separately transformed into CB15N in order to insert *chi* forward and reverse sites after CCNA_00774 or CCNA_00836. These were then transduced into ML2464 to give ML2467-2470.

For ML2471, first ML2461 was transformed into ML743 [5] to insert a *gent* resistance cassette near the *recA* locus. A lysate of this strain was used to transduce *ΔrecA* into ML2464 to give ML2471.

For ML2425, ML2447 and ML2643, first ML2648-2503 were separately transformed into CB15N in order to insert P_{recA}-*recA* (wild type and mutants) at the *xyl* locus. These were then transduced into ML2471 to give ML2425, ML2447 or ML2643.

For ML2644, ML2091 [7] was transduced into ML2464 to give ML2644.

Plasmid construction

For ML2647, the last ~600bp of CCNA_03821 were amplified using oligos Isce1_ccna03821_up_forward and Isce1_ccna03821_up_reverse. *tet* resistance cassette was amplified using oligos tet_forward and tet_reverse and ~600bp downstream of CCNA_03821 was amplified using oligos Isce1_ccna03821_down_forward and Isce1_ccna03821_down_reverse. These fragments were combined by SOE PCR using oligos Isce1_ccna03821_up_forward and Isce1_ccna03821_down_reverse. The resultant fragment was digested with BamH1 and Nhe1 and cloned into BamH1-Nhe1 digested pNPTS138. The plasmid was checked by sequencing for the correct insert.

For ML2648, P_{recA}-*recA* was amplified using oligos P_{recA}-*recA*_forward and P_{recA}-*recA*_reverse. The fragment was cloned into NdeI-NheI digested pMT697. The plasmid was checked by sequencing.

For ML2649, oligos $RecA^{KA}$_up and $RecA^{KA}$_down were used to carry out site-directed-mutagenesis on ML2648. The plasmid was checked by sequencing.

For ML2650-ML2653, first the *chi* inserts (forward and reverse) were PCR amplified using oligos chi^{For}_forward and chi^{For}_reverse or chi^{Rev}_forward and chi^{Rev}_reverse. Separately, ~800 bp after CCNA_00774 or CCNA_00836 were PCR ampflied using oligos ccna_00774_forward and ccna_00774_reverse or ccna_00836_forward and ccna_00836_reverse. The two fragments were assembled into pMT635 using Gibson assembly cloning.

For bacterial two hybrid plasmids (ML2654-ML2660), *recA, addA* and *addB* were amplified using B2h forward and reverse oligos and cloned into BamHI-KpnI digested pKT25 or pUT18C plasmids. The resulting constructs were verified by sequencing.

Supporting Results

Kinetic modeling of the AddAB DNA degradation process

We constructed a four-step model to simulate the kinetics of DNA degradation/ processing by the AddAB complex in a single cell. Our model is informed partly by *in vitro* biochemical experiments on AddAB activity [8–10]. Custom code to simulate DSBs and AddAB degradation was written in MATLAB. Simulations were run with 1 kb resolution for a population of 10,000 cells.

1. AddAB binding

To calculate rate of AddAB binding or DSB formation (t_{DSB}), we tested three possibilities for the time it takes from enzyme induction (t_{ind}) to t_{DSB}: a. After induction, there is a constant rate of DSB formation. b. After induction, the waiting times for DSB formation is exponentially distributed, with a peak in DSB formation soon after induction of enzyme. c. After induction, there is a burst of DSB induction at a particular time (i.e. most cells have a DSB within a short time window). We then searched for rates for all three scenarios that would closely reflect the depth and pattern of the *in vivo* degradation profiles. While the burst scenario did not match any profiles, we found that constant rates of DSB formation as well as exponentially distributed waiting times could both closely recapitulate the 1 h *in vivo* DNA degradation profiles. However, since the fold change in CFU (Fig S1H) and the depth change in the *in vivo* profiles at later time points reflects an almost linear effect of DSB induction over time, we assumed that upon induction, DSB at a specific site occurs with equal probability at any time point between t_{ind} and t_{max} (maximum time taken for all cells to have a DSB) = 8550 – 10300s (142 – 172min) (Fig S4A-B). The exact value was adjusted for each experimental condition to match the depth of the *in vivo* degradation profiles. These rates were also measured experimentally via qPCR (Fig S4 C-D) and matched closely the rates predicted from simulations.

2. Initial DNA degradation

Degradation was simulated at a resolution of 1 kb. Processing times for each segment of 1 kb were drawn from an exponential distribution with mean τ_0 = 2.5 ms/bp corresponding to a degradation rate of $k_0 = 1/\tau_0$ = 400 bp/s.

3. *chi* recognition

chi recognition was modeled as a stochastic process. Sites were recognized with probability p*chi* and recognition led to a reduced degradation rate of k_{slow} (also referred to as post-*chi* AddAB). Processing times after *chi* recognition were rescaled by a factor k_{slow}/k_0 to account for the reduced degradation/ processing rate. If AddAB dissociated from DNA before *chi* recognition, it was assumed to immediately reload and continue DNA degradation.

4. AddAB dissociation

We also introduced the possibility for AddAB to dissociate from the DNA after *chi* recognition. Again, this step was modeled as a Poisson process with exponentially distributed waiting times and a rate constant k*Off*. Processing times for 1 kb segments after dissociation were set to infinity.

We determined degradation times for 1 kb segments across the entire length of the genome. The cumulative sum of degradation times from the break site up to a specific 1 kb segment represents the AddAB 'travel time' to that position. Experimentally, DNA degradation was allowed to occur for a fixed period of time. Therefore, we set a threshold of t_{max} corresponding to the duration of DSB induction. All DNA with a travel time shorter than t_{max} was considered degraded in our simulation. Thus, for each cell we obtained a binary trace indicating which stretch of DNA had been degraded. To model the results of a sequencing experiment we averaged 10,000 binary traces. For overlay of simulated and experimental degradation profiles, see Fig S5.

Scanning for best parameter sets

We sought to find the parameter sets (p*chi*, k_{slow}, k*Off*) that best describe the experimentally obtained degradation profiles. In lieu of an analytical solution we

sampled the parameter space by varying independently p*chi* = 0.10 – 0.41 in steps of 0.01, k_{slow} = 10 – 400 bp/s in steps of 10 bp/s, and k*Off* = 0 – 10x10^{-3}/s in steps of 0.1x10^{-3}/s to find parameters that best describe experimental results. The 'goodness of fit' of a specific parameter set was assessed by calculating the root-mean-squared deviation (RMSD) between the simulated average trace and experimental results in an 800 kb window around the break site. This procedure was repeated for double strand breaks introduced a two different positions in the genome (+780 kb, +3042 kb) and durations of DSB induction t_{max} = 1 h and 2 h respectively.

We first tested a scenario where we varied k_{slow} and p*chi* without considering the possibility of k*Off* (k*Off* = 0). We found that a parameter set with pre-*chi* AddAB speed of 400 bp/s, p*chi* of 0.22 (CI 0.192-0.242) and k_{slow} of 51 bp/s (CI 46-56 bp/s) successfully described the experimental data for double strand breaks introduced at +780 kb and +3042 kb for 1 h or 2 h. However, this scenario did not successfully match the shape of the degradation profile 4 h after DSB induction (Fig S5).

We then considered the parameter of k*Off* as well. In particular, we tested for two distinct models of AddAB action:

- Model 1, in which the processing rate after *chi* recognition was set to a value obtained in *in vitro* experiments (fixed k_{slow} = 340 bp/s);
- and a general Model 2, in which all three parameters were varied.

The parameter sets with the smallest average RMSD for all experimental conditions combined and for the different models are listed below.

Simulation	Pre-*chi* AddAB	p*chi*	Post-*chi* AddAB (k*chi*)	k*Off* (1/s)	RMSD				
					780 kb (1 h)	780 kb (2 h)	3042 kb (1 h)	3042 kb (2 h)	average
Model 1	400 bp/s	0.25	340 bp/s	0.0017	0.000239	0.002429	0.000657	0.000832	0.001055
Model 2	400 bp/s	0.23	100 bp/s	0.0003	0.000335	0.000802	0.000456	0.000505	0.000524

Refined parameter sets and uncertainties

The parameter scan yields discrete values for (p*chi*, k_{slow}, k*Off*). To obtain refined values and estimate uncertainties for each parameter we performed 3rd order polynomial fits

around the minimum in the RMSD as a function of the respective parameter. The fit returns the best guess for each parameter in both models. By running 1000 independent simulations with the newly obtained sets of best parameters we obtained a distribution of RMSD values for the respective parameter sets. An RMSD threshold was defined such that 95% of the simulation runs had an RMSD smaller than this value (one-sided test). Uncertainties (95% confidence intervals) are given by parameter values for which the fit exceeds the RMSD threshold (Fig S6 and below).

Simulation	Pre-*chi* AddAB (k_0)	p*chi*	Post-*chi* AddAB (k_{slow})	k*Off* (1/s)
Model 1	400 bp/s	0.233 [0.203-0.267]	340 bp/s	0.0017 [0.0014-0.002]
Model 2	400 bp/s	0.236 [0.205-0.276]	96 bp/s [84-109 bp/s]	0.00035 [0.00026-0.00045]

Predicting focus loss

Models 1 and 2 match the all datasets equally well. However, the degradation rate after *chi* recognition varies significantly between the two models. We therefore predicted lag times between the degradation of genomic loci flanking a 100 or 200 kb stretch rich in *chi* sites. These loci were fluorescently labeled and fluorescence was lost upon DNA degradation. We expect that after passing through a larger number of *chi* sites, each with a recognition probability of ~0.23, most molecules will have slowed down to k_{slow} (post-*chi* AddAB) from the initial degradation rate k_0 (pre-*chi* AddAB). Predictions for the lag time between the loss of the two foci can be extracted from our simulations. The most likely lag times resulting from Model 1 are 4 min for a stretch of 100 kb and 9 min for 200 kb; and 15 min or 30 min for the same stretches according to Model 2. Experimental results yielded mean values very close to the values obtained for Model 2 (see Fig 4).

Supporting References

1. Badrinarayanan A, Le TBK, Laub MT. Rapid pairing and resegregation of distant homologous loci enables double-strand break repair in bacteria. J Cell Biol. 2015;210: 385–400. doi:10.1083/jcb.201505019

2. Aakre CD, Phung TN, Huang D, Laub MT. A bacterial toxin inhibits DNA replication elongation through a direct interaction with the β sliding clamp. Mol Cell. 2013;52: 617–628. doi:10.1016/j.molcel.2013.10.014

3. Skerker JM, Prasol MS, Perchuk BS, Biondi EG, Laub MT. Two-component signal transduction pathways regulating growth and cell cycle progression in a bacterium: a system-level analysis. PLoS Biol. 2005;3: e334. doi:10.1371/journal.pbio.0030334

4. Thanbichler M, Iniesta AA, Shapiro L. A comprehensive set of plasmids for vanillate- and xylose-inducible gene expression in *Caulobacter crescentus*. Nucleic Acids Res. 2007;35: e137. doi:10.1093/nar/gkm818

5. Modell JW, Hopkins AC, Laub MT. A DNA damage checkpoint in *Caulobacter crescentus* inhibits cell division through a direct interaction with FtsW. Genes Dev. 2011;25: 1328–1343. doi:10.1101/gad.2038911

6. Ely B. Genetics of *Caulobacter crescentus*. Methods Enzymol. 1991;204: 372–384. doi:10.1016/0076-6879(91)04019-K

7. Modell JW, Kambara TK, Perchuk BS, Laub MT. A DNA damage-induced, SOS-independent checkpoint regulates cell division in *Caulobacter crescentus*. PLoS Biol. 2014;12: e1001977. doi:10.1371/journal.pbio.1001977

8. Carrasco C, Gilhooly NS, Dillingham MS, Moreno-Herrero F. On the mechanism of recombination hotspot scanning during double-stranded DNA break resection. Proc Natl Acad Sci. 2013;110: E2562–E2571. doi:10.1073/pnas.1303035110

9. Gilhooly NS, Dillingham MS. Recombination hotspots attenuate the coupled ATPase and translocase activities of an AddAB-type helicase-nuclease. Nucleic Acids Res. 2014;42: 5633–5643. doi:10.1093/nar/gku188

10. Gilhooly NS, Carrasco C, Gollnick B, Wilkinson M, Wigley DB, Moreno-Herrero F, et al. *Chi* hotspots trigger a conformational change in the helicase-like domain of AddAB to activate homologous recombination. Nucleic Acids Res. 2016;44: 2727–2741. doi:10.1093/nar/gkv1543
